# A large and functionally diverse family of *Fad2* genes in safflower (*Carthamus tinctorius* L.)

**DOI:** 10.1186/1471-2229-13-5

**Published:** 2013-01-07

**Authors:** Shijiang Cao, Xue-Rong Zhou, Craig C Wood, Allan G Green, Surinder P Singh, Lixia Liu, Qing Liu

**Affiliations:** 1 Commonwealth Scientific and Industrial Research Organization Plant Industry, PO Box 1600, Canberra, ACT 2601, Australia; 2 School of Life Sciences, Northeast Normal University, Changchun, China

## Abstract

**Background:**

The application and nutritional value of vegetable oil is highly dependent on its fatty acid composition, especially the relative proportion of its two major fatty acids, i.e oleic acid and linoleic acid. Microsomal oleoyl phosphatidylcholine desaturase encoded by *FAD2* gene is known to introduce a double bond at the Δ12 position of an oleic acid on phosphatidylcholine and convert it to linoleic acid. The known plant FAD2 enzymes are encoded by small gene families consisting of 1-4 members. In addition to the classic oleate Δ12-desaturation activity, functional variants of FAD2 that are capable of undertaking additional or alternative acyl modifications have also been reported in a limited number of plant species. In this study, our objective was to identify *FAD2* genes from safflower and analyse their differential expression profile and potentially diversified functionality.

**Results:**

We report here the characterization and functional expression of an exceptionally large *FAD2* gene family from safflower, and the temporal and spatial expression profiles of these genes as revealed through Real-Time quantitative PCR. The diversified functionalities of some of the safflower *FAD2* gene family members were demonstrated by ectopic expression in yeast and transient expression in *Nicotiana benthamiana* leaves. *CtFAD2-1* and *CtFAD2-10* were demonstrated to be oleate desaturases specifically expressed in developing seeds and flower head, respectively, while *CtFAD2-2* appears to have relatively low oleate desaturation activity throughout the plant. *CtFAD2-5* and *CtFAD2-8* are specifically expressed in root tissues, while *CtFAD2-3, 4, 6, 7* are mostly expressed in the cotyledons and hypocotyls in young safflower seedlings. *CtFAD2-9* was found to encode a novel desaturase operating on C16:1 substrate. CtFAD2-11 is a tri-functional enzyme able to introduce a carbon double bond in either *cis* or *trans* configuration, or a carbon triple (acetylenic) bond at the Δ12 position.

**Conclusions:**

In this study, we isolated an unusually large *FAD2* gene family with 11 members from safflower. The seed expressed *FAD2* oleate Δ12 desaturase genes identified in this study will provide candidate targets to manipulate the oleic acid level in safflower seed oil. Further, the divergent FAD2 enzymes with novel functionality could be used to produce rare fatty acids, such as crepenynic acid, in genetically engineered crop plants that are precursors for economically important phytoalexins and oleochemical products.

## Background

Safflower (*Carthamus tinctorius* L.) is an ancient oilseed crop that is currently grown for its high quality edible oil used in cooking, salad dressings and margarines, and to a lesser degree as a bird seed. The characteristics of oils are highly dependent on their fatty acid composition. Oleic acid (C18:1^Δ9^) and linoleic acid (C18:2^Δ9,12^) are the two major fatty acids found in safflower seed oil, together accounting for about 90% of the total fatty acids. Conventional safflower oil is characterised by its relatively high level of linoleic acid content about 70% compared to most other oilseed crops [1]. In the past six decades, breeders have exploited safflower’s natural genetic diversity to modify the oleate/linoleate ratio for particular end use purposes. Numerous breeding lines with high levels of either oleic acid (75-84%) or linoleic acid (71-89%) have been selected. The relative proportions of these two major fatty acids determine relevant technological and nutritional properties of edible oils [2]. Nutritionally, both oleic acid and linoleic acid can lower total serum cholesterols, however, oleic acid has higher oxidative stability compared to linoleic acid as it contains one less double bond. Therefore raising oleic acid content at the expense of linoleic acid has been set as an important research objective for the improvement of many oilseed crops, including safflower, to provide highly stable cooking oils without the need for hydrogenation, a process that can result in the formation of nutritionally undesirable *trans* fatty acid [3,4]. Beyond food applications, high oleic oils also have significant existing and potential industrial uses, such as in improved biodiesel, lubricants, and hydraulic oils because of the high oxidative stability needed in these products. Purified oleic acid is also a valuable industrial chemical feedstock, and can be cleaved to form derivatives such as azelaic acid that can be used in the formulation of a range of industrial products and polymers [5-7].

The distinct fatty acid compositions found in seed storage oil and membrane lipids are the result of an intricate metabolic network that regulates fatty acid biosynthesis and flux through both prokaryotic and eukaryotic pathways [8,9]. It is understood that the chloroplast Δ12-desaturase (FAD6) is necessary for desaturating 16:1 and 18:1 fatty acids to 16:2 and 18:2 on all 16:1- or 18:1-containing chloroplast membrane lipids including phosphatidyl glycerol (PG), monogalactosyldiacylglycerol (MGDG), digalactosyldiaclyglycerol (DGDG), and sulfoguinovosyldiacylglycerol (SQDG) [9]. The enzyme primarily responsible for the synthesis of linoleic acid from oleic acid in seed storage lipids is the microsomal oleoyl phosphatidylcholine desaturase (FAD2) that introduces a double bond at the Δ12 position of oleic acid on phosphatidylcholine (PC), forming linoleic acid on the endoplasmic reticulum (ER) [10-12]. Variants of the FAD2 enzyme are also known to have diversified functionalities in fatty acid modification, catalysing hydroxylation [13,14], epoxidation [15], and the formation of acetylenic bonds [15,16] and conjugated double bonds [17-20]. Some functionally divergent FAD2 enzymes are multi-functional, such as the bifunctional hydroxylase/desaturase from *Lesquerella fendleri*[21], and tri-functional acetylenase from *Crepis alpina*, which can also catalyse the formation of both *trans* and *cis* double bonds at the Δ12 position of oleic acid [22].

Membrane integrity and function, determined by structure and fluidity, are largely affected by lipid composition and the degree of fatty acid desaturation in plants [23]. Because the FAD2 enzyme is the key step in the accumulation of polyunsaturated fatty acids it plays an essential role in the biophysical characteristics of cell membranes and is often induced in response to various environmental stimuli such as extreme temperatures [8], high salt [24], and pathogen attack [25]. The expression of FAD2 leading to the production of polyunsaturated fatty acids is also important to the specific signal transduction pathways, such as jasmonic acid pathway that is known to be a critical factor in plant defence system and male fertility [26-28].

Since the cloning of first plant *FAD2* gene in *Arabidopsis thaliana*[11], its orthologous DNA sequences have been isolated and characterised from many different plant species, including soybean [29], rape [30], cotton [31-34], peanut [35,36] and flax [37]. Only a single *FAD2* gene exists in Arabidopsis, but in most other plant species FAD2 is encoded by small gene families. For example, FAD2 is encoded by three distinct family members in soybean [29,38] and by four members in cotton [31-34]. Here we report the discovery, isolation and characterisation of an unprecedentedly large *FAD2* gene family from safflower. Phylogenetic analysis of eleven full length cDNAs and their distinct genomic structural features indicated that they are non-allelic and have likely evolved through gene duplication at several hierarchical levels. Their distinct expression patterns were revealed by real time quantitative PCR (RT-qPCR) of different safflower tissues. Functional divergence of the FAD2 family members was explored by heterologous expression in yeast and transient expression in *Nicotiana benthamiana*.

## Results

### Cloning and sequencing analysis of multiple members of safflower *CtFAD2* gene family

Two different full length cDNAs, designated as *CtFAD2-1* and *CtFAD2-2*, were isolated from the lambda cDNA library derived from safflower developing embryos, using Arabidopsis *FAD2* DNA sequence as a probe. The Arabidopsis *FAD2* DNA sequence was also used to “blast” search the Expressed Sequence Tags (ESTs) database generated by the Compositae Genome Project (CGP, http://compgenomics.ucdavis.edu/compositae_index.php). From the total 41,317 safflower ESTs at the time we searched, at least eleven distinct *FAD2* cDNA sequence contigs were identified and designated. In addition to *CtFAD2-1* and *CtFAD2-2* that had already been isolated from the developing embryo cDNA library, a further 9 partial cDNAs were designated as *CtFAD2-3* through to *CtFAD2-11,* respectively. Gene specific primers were designed from each of the partial cDNA sequences, and the full length cDNA were obtained by conducting 3^′^ and 5^′^ rapid amplification of cDNA ends (RACE) reaction on RNAs derived from various safflower plant tissues, including leaf, root, hypocotyl and flower head. The amplified fragment was subcloned into the pGEM-Teasy vector and sequenced from both directions. Sequence comparisons of the 3^′^ and 5^′^ ends with the corresponding ESTs showed overlapping regions that matched with each other. Subsequently full length cDNAs encoding the 9 distinct partial *FAD2* ESTs were obtained by assembling the RACE product with corresponding ESTs. Each cDNA contains an uninterrupted coding region that shares extensive sequence homology with each other, and unique 5^′^ and 3^′^ untranslated region (UTR) sequences. The putative polypeptides were varied between 372-388 amino acids. The sequence similarity among these 11 CtFAD2 coding regions at both nucleotide and amino acid levels is listed in Table 1. It was also revealed that the putative polypeptides of the 11 *CtFAD2* genes share about 50%-65% sequence identity to orthologous FAD2 enzymes in other plants.

**Table 1:** **Sequence identity of the coding region DNA and deduced amino acids in safflower *CtFAD2 *genes**

			**Deduced amino acids**									
		***CtFAD2-1***	***CtFAD2-2***	***CtFAD2-3***	***CtFAD2-4***	***CtFAD2-5***	***CtFAD2-6***	***CtFAD2-7***	***CtFAD2-8***	***CtFAD2-9***	***CtFAD2-10***	***CtFAD2-11***
**Nucleotide acid**	*CtFAD2-1*	-	70.3%	53.2%	52.5%	53.5%	50.9%	54.1%	59.7%	59.5%	80.1%	56.4%
	*CtFAD2-2*	70.0%	-	54.5%	55.0%	54.2%	51.7%	57.8%	60.6%	62.5%	69.5%	58.6%
	*CtFAD2-3*	62.0%	62.0%	-	97.1%	62.0%	61.8%	63.1%	52.7%	50.9%	51.2%	56.8%
	*CtFAD2-4*	62.7%	63.3%	95.1%	-	61.4%	61.4%	63.3%	53.1%	50.9%	50.9%	56.9%
	*CtFAD2-5*	61.9%	60.3%	69.7%	70.6%	-	63.2%	62.0%	51.4%	51.3%	51.7%	53.9%
	*CtFAD2-6*	60.6%	59.9%	68.8%	69.6%	72.0%	-	63.1%	49.3%	50.8%	50.1%	56.2%
	*CtFAD2-7*	62.2%	65.8%	69.4%	69.3%	66.6%	68.2%	-	51.7%	49.2%	51.4%	60.7%
	*CtFAD2-8*	65.2%	66.2%	63.1%	62.8%	60.8%	61.7%	61.2%	-	58.8%	58.1%	56.40%
	*CtFAD2-9*	64.9%	66.2%	59.5%	59.5%	58.3%	59.2%	59.5%	63.5%	-	59.3%	55.9%
	*CtFAD2-10*	78.9%	72.0%	60.7%	62.0%	59.8%	61.0%	60.7%	64.3%	64.1%	-	57.2%
	*CtFAD2-11*	60.0%	62.9%	64.1%	64.4%	62.4%	64.1%	62.7%	63.9%	60.9%	61.7%	-

**Figure 1:**
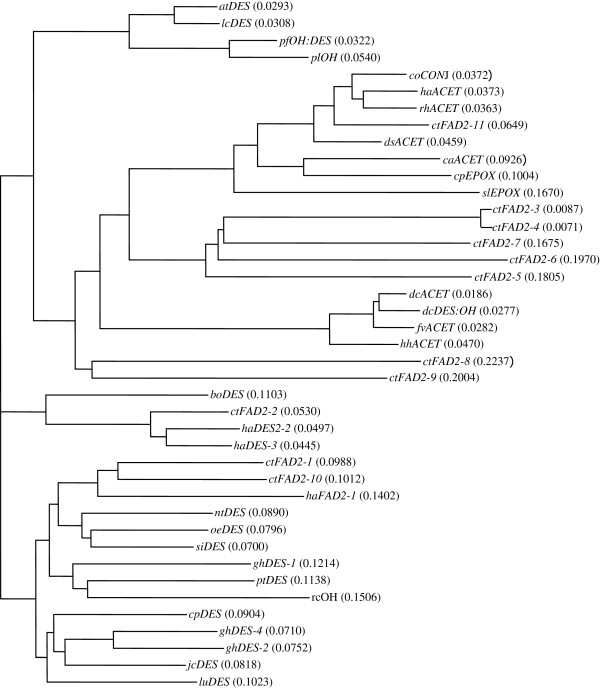
**Phylogenetic comparison of safflower *CtFAD2 *gene family and orthologous FAD2s from other plants.** The phylogenetic tree was generated by Vector NTI (invitrogen). Included in the alignment were FAD2 desaturases (DES), hydroxylases (OH), epoxygenases (EPOX), acetylenases (ACET), and conjugases (CONJ). The GenBank accession numbers of the amino acid sequences represented in the phylogenetic tree are: *atDES*, AAM61113.1; *lcDES*, ACR15954.1; *pfOH:DES*, AAC32755.1; *plOH*, ABQ01458.1; *coCONJ*, AAK26632.1; *haACET*, ABC59684.1; *rhACET*, AAO38035.1; *dsACET*, AAO38036.1; *caACET*, ABC00769.1; *cpEPOX*, CAA76156.1; *slEPOX*, AAR23815.1; *dcACET*, AAO38033.1; *dcDES:OH*, AAK30206.1; *fvACET*,AAO38034.1; *hhACET*, AAO38031.1; *boDES*, AAC31698.1; *haDES-2*, AAL68982.1; *haDES-3*, AAL68983.1; *haDES-1*, AAL68981.1; *ntDES*, AAT72296.2; *oeDES*, AAW63040; *siDES*, AAF80560.1; *ghDES-1*, CAA65744.1; *ptDES*, XP_002297660.1; *rcOH*, AAC49010.1; *cpDES*, AAS19533.1; *ghDES-4*, AAQ16653.1; *ghDES-2*, CAA71199.1; *jcDES*, ADB93805.1; *luDES*, ACF49507.1. (at, *Arabidopsis thaliana*; lc, *Lepidium campestre*; pf, *Physaria fendleri*; pl, *Physaria lindheimeri*; co, *Calendula officinalis*; ha, *Helianthus annuus*; rh, *Rudbeckia hirta*; ds, *Dimorphotheca sinuate*; ca, *Crepis alpine*; cp, *Crepis palaestina*; sl, *Stokesia laevis*; dc, *Daucus carota*; fv, *Foeniculum vulgare*; hh, *Hedera helix*; Bo, *Borago officinalis*; nt, *Nicotiana tabacum*; oe, *Olea europaea*; si, *Sesamum indicum*; gh, *Gossypium hirsutum*; pt, *Populus trichocarpa*; rc, *Ricinus communis*; cp, *Cucurbita pepo*; jc, *Jatropha curcas*; lu, *Linum usitatissimum*).

To elucidate phylogenetic relationship of safflower *FAD2* genes, the 11 deduced polypeptide sequences were aligned with orthologous FAD2 sequences and a neighbour-joining tree was constructed using Vector NTI. As shown in Figure 1, CtFAD2-1 and CtFAD2-10 are aligned next to each other and to other seed expressed FAD2s, such as sunflower HaFAD2-1 and cotton GhFAD2-1. CtFAD2-2 is aligned together with other constitutively expressed genes, such as sunflower HaFAD2-2 and HaFAD2-3. CtFAD2-3, 4, 5, 6 and 7 form a new branch, most likely to have recently become divergent. Interestingly it is embedded deep in a clade with functionally divergent FAD2 fatty acid modifying enzymes, such as fatty acid acetylenases and epoxygenases. CtFAD2-11 is also aligned together with fatty acid acetylenases from several plant species, including the sunflower vFAD2 that was induced by fungal elicitors [39]. 

The alignment of the putative polypeptide sequences of CtFAD2s together with the selected plant orthologs is given in (Additional file 1: Figure S1). Similar to other plant FAD2, the putative polypeptides of CtFAD2s contain an aromatic amino acid-rich motif, at the C-terminus, which is both necessary and sufficient for maintaining localization in the ER [40]. Consistent with other plant membrane bound fatty acid desaturase enzymes, the putative polypeptides deduced from all eleven safflower *CtFAD2* cDNAs contain three highly conserved histidine-rich motifs that have been implicated in the formation of the diiron-oxygen complex used in biochemical catalysis [41]. The first histidine motif is HECGHH in majority of CtFAD2 putative polypeptide sequences, except CtFAD2-5 and -6 that have HDCGHH, HDLGHH, respectively. The last amino acid His (H) has been converted to a Gln (Q) in CtFAD-8 (HECGHQ). We have compared this motif in 55 plant FAD2 enzymes and the H to Q substitution is also present in a diverged FAD2 homologue from *Lesquerella lindheimeri* with predominantly fatty acid hydroxylase activity [42]. The second histidine motif is highly conserved as HRRHH in several safflower FAD2s, including CtFAD2-1, 2, 8, 9 and 10. It is noteworthy that at +3 of the motif an Asn (N) substitution was found in CtFAD2-11, consistent with a number of functionally divergent FAD2s, including *Crepis alpina* CREP1 (ABC00769), *C. palaestina* Cpal2 (CAA76156) and sunflower vFAD2 (AY166773.1), *Calendula officinalis* FAC2 (AF343064.1), *Rudbeckia hirta* acetylenase (AY166776.1). Either a Ser (S) or Thr (T) substitution spotted at this position in CtFAD2-3, 4, 5, 6 and 7.

In CtFAD2-1, 2, 9, 10 the amino acid immediately preceding the first histidine box is an Ala (A), consistent with other plant FAD2 Δ12 oleate desaturase enzymes. Ala substitution by Val (V) occurred in CtFAD2-5*,* while other six CtFAD2 enzymes have Gly (G) in this position. It was proposed by Cahoon *et al.*[39] that Gly substitution of Ala at this position has been consistently found in functionally divergent FAD2 enzymes, except Δ12 hydroxylase. It is noted that only CtFAD2-11 has a DVTH motif, in the -5 to -2 positions of the third Histidine box, which fits with the (D/N)VX(H/N) motif proposed to occur in all plant acetylenases [43]. The five amino acids immediately behind the third histidine box of CtFAD2-1, 2, 10, are LFSTM as for other known plant FAD2 Δ12 oleate desaturases. In contrast, CtFAD2-9 has an LFSYI motif at this position with two amino acid substitutions at +4 and +5 position. In CtFAD2-3, 4 and 7, the S at the +3 position is substituted by Pro (P), which is also exclusively present in other FAD2 fatty acid conjugases, including those from *Calendula officinalis* (FAC2, Genbank AAK26632) and *Trichosanthes kirilowii* (Genbank AAO37751).

It was shown that the Serine-185 of soybean seed-specific GmFAD2-1 sequences is phosphorylated during seed development [44], as a regulatory mechanism for its enzymatic activity. Among the 11 safflower FAD2 enzymes only CtFAD2-1, the seed-specific FAD2, has the Serine corresponding with that in soybean FAD2-1. It is tempting to predict that the same posttranslational regulatory mechanism of *FAD2* expression through phosphorylation of target site including the Serine-185 may play an important role in modulating microsomal Δ12 oleate desaturation during safflower seed development and oil accumulation.

### Genomic structures and evolutionary studies of the *CtFAD2* gene family in safflower

In the context of elucidating the gene structure of *FAD2* from safflower, an intron that is situated in the 5^′^ UTR of *FAD2* genes was isolated. From each of the 11 safflower *FAD2* cDNA sequences, the typical splice site (AG:GT) was predicted and PCR primers were designed for amplification of such an intron from genomic DNA. The predicted 5^′^ intron was obtained from 8 of the 11 *CtFAD2* genes, including *CtFAD2-1, -2, -3, -4, -5, -7, -10* and *-11*. The major features of the primary DNA sequences of these *CtFAD2* introns are summarised in Table 2. The intron was not amplified from *CtFAD2-6, -8* and *-9*, likely because the 5^′^ UTR in which the introns reside is incomplete in our clones. All of the eight intron sequences were located within the 5^′^ UTR, at positions that varied between 11 to 38 bp upstream of the putative start codon, the first ATG. The intron length varied between 114 to 3,090 bp and was 1,144 bp for *CtFAD2-1*, similar in size to the introns identified in *FAD2* genes from Arabidopsis (The Arabidopsis Information Resource, http://www.arabidopsis.org), cotton [45] and sesame (*Sesamum indicum*) [46]. The variations in the relative positions and the substantial differences in the sizes of the 5^′^-UTR introns are distinguishing structural differences among safflower *FAD2* genes, which could be important in differential expression of the genes. The large intron present in the 5^′^-UTR of *FAD2* genes could play an important role in expression regulation, as it has been reported to have positive effects on the expression of reporter genes in sesame [46]. The equivalent intron has also been shown to be an effective target for posttranscriptional gene silencing of *FAD2* in soybean [47]. 

**Table 2:** **The features of *CtFAD2 *gene introns**

	**Position**	**Length**	**AT content**	**CG content**	**5`E/I boundary**	**3`I/E boundary**
*CtFAD2-1*	-13	1144	64.5%	35.5%	AG:GTGCAT	TTGCAG:GT
*CtFAD2-2*	-12	3090	65.8%	34.2%	AG:GTGAGA	TTGCAG:GT
*CtFAD2-3*	-11	114	73.7%	26.3%	AG:GTATGA	ATGCAG:GT
*CtFAD2-4*	-11	124	75.0%	25.0%	AG:GTAAGT	GCGCAG:GT
*CtFAD2-5*	-33	96	69.8%	30.2%	AG:GTACCT	TTTCAG:GT
*CtFAD2-7*	-29	242	61.6%	38.4%	AG:GTATAC	TTGCAG:GT
*CtFAD2-10*	-38	2247	68.9%	31.1%	TG:GTTCGT	TTACAG:GT
*CtFAD2-11*	-22	334	62.5%	37.5%	AG:CTCACA	GTCTTT:GT

The putative splice sites, at both 5^′^ exon/intron and 3^′^ intron/exon, are conserved as AG:GT in most of the eight *CtFAD2* genes, except the 5^′^ exon/intron (TG:GT) in *CtFAD2-10*, and both the 5^′^ exon/intron (AG:CT) and 3^′^ intron/exon (TT:GT) in *CtFAD2-11*. However, the intron sequences themselves are all highly divergent without any significant homology among them. These intron sequences are AT-rich with AT content between 61% and 75%, consistent with other dicot intron sequences. Analyzing the intron sequences by the PLACE program (http://www.dna.affrc.go.jp/PLACE/) identified several putative cis-regulatory elements. For instance, a few motifs, such as ABRE and SEF4, commonly present in seed-specific promoters were located in the seed-specific *CtFAD2-1*. Likewise, an AG-motif that is often present in the promoter of defence-related genes induced by various stresses, such as wounding or elicitor treatment, was located in the *CtFAD2-3* gene that is specifically expressed in the hypocotyls and cotyledons of young safflower seedlings.

### Analysis safflower *CtFAD2* gene family by Southern blot analysis

**Figure 2:**
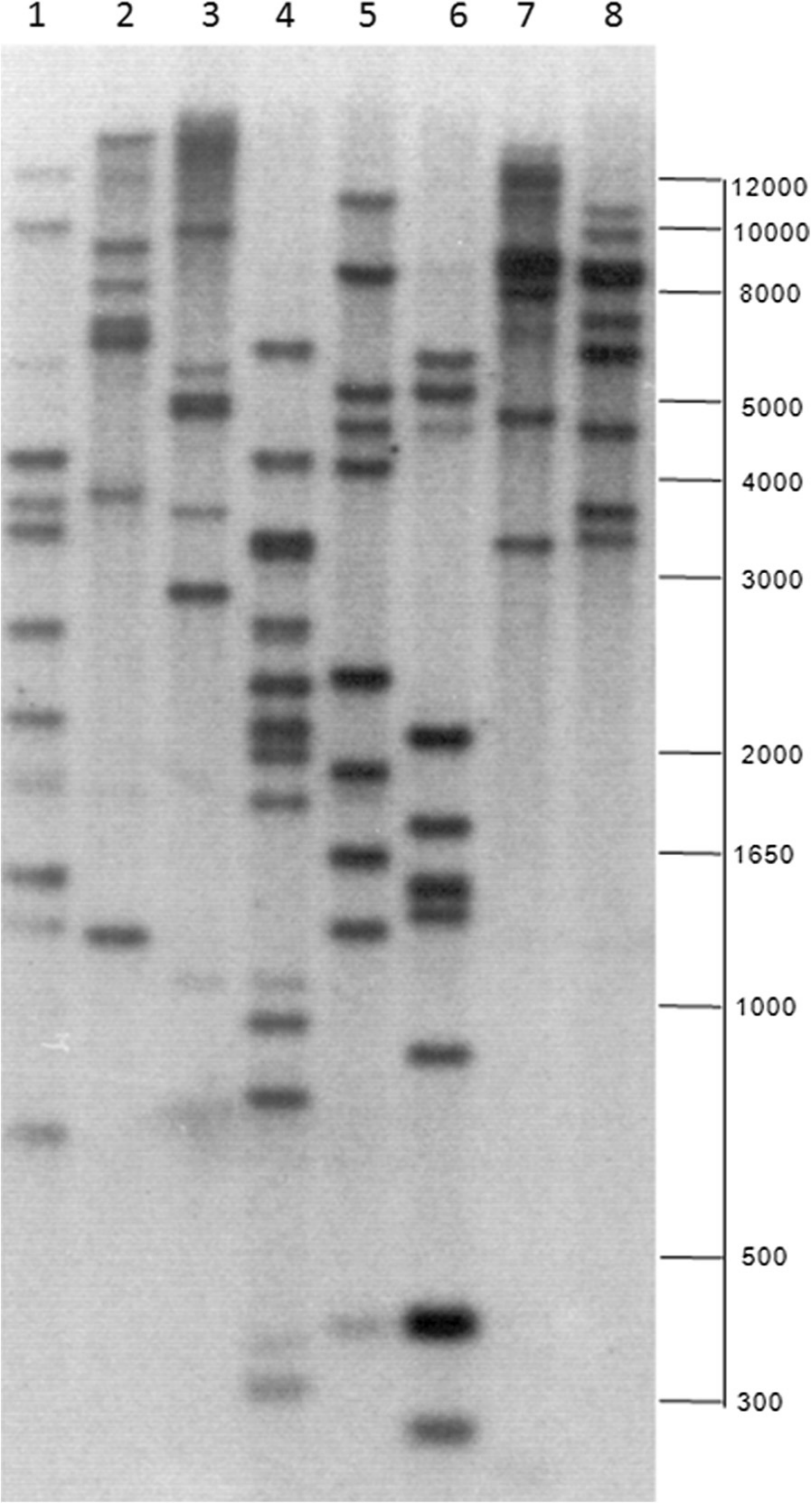
**Southern blot analysis of genomic structure in safflower genotype SU.** Genomic DNA samples were digested with eight different restriction enzymes prior to separation on agarose gel. These enzymes include *Acc*I (1), *Bgl*II (2), *Bam*HI (3), *Eco*RI (4), *Eco*RV (5), *Hin*dIII (6), *Xba*I (7) and *Xho*I (8). The blot was probed with radio-labeled entire coding region of *CtFAD2-6* and washed at low stringency conditions.

The complexity of the *FAD2* gene family in safflower was also examined by low stringency Southern blot analysis and confirmed that safflower FAD2 is encoded by a complex multigene family (Figure 2). The restriction fragment patterns derived from various restriction enzymes are consistent with there being more than 10 *FAD2* genes in safflower. The variation in the intensity of hybridization signals reflects the relative levels of sequence similarity with the probe DNA used.

### Expression profile of *CtFAD2* genes in different tissues

To determine tissue expression patterns of the various *CtFAD2* genes, RT-PCR analysis was carried out with total RNA extracted from cotyledon, hypocotyl, root and leaf tissues derived from young seedlings, as well as flower tissues and developing embryos of safflower genotype SU. No product was detectable after 40 cycles of amplification in control reactions without reverse transcriptase, demonstrating the absence of genomic DNA in RNA samples.

**Figure 3:**
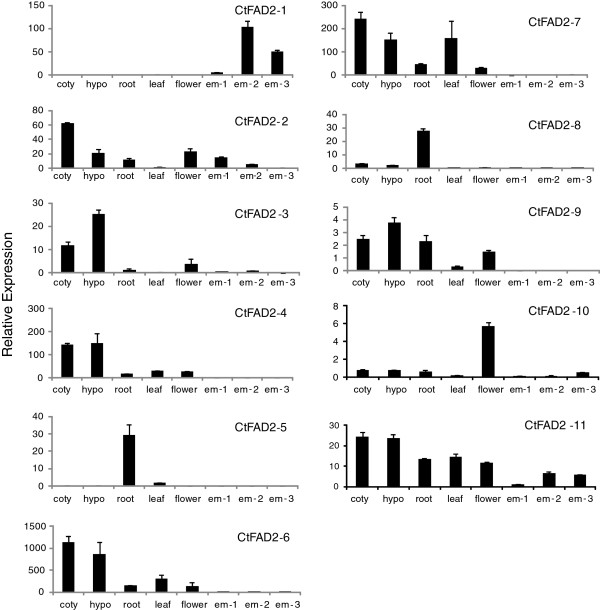
**RT-qPCR expression analysis of 11 safflower *CtFAD2 *genes in various safflower plant tissues, including cotyledon (coty), hypocotyls (hypo), root, leaf, flower and immature embryos of three progressing developmental stages, early (em-1), middle (em-2) and late (em-3).**

As shown in Figure 3, *CtFAD2-1* is specifically expressed in developing seeds, with little if any expression detected in the somatic tissues examined. *CtFAD2-2* is expressed at low levels in seeds as well as in all other tissues examined. *CtFAD2-4, -5, -6, -7, -8, -9* showed no expression in developing embryos. Low, yet detectable levels of *CtFAD2-10* and -11 expression were observed in developing seeds, especially in the late development stage. *CtFAD2-4, -6, -7, -9* and *-11* showed high expression in young seedling tissues including cotyledons and hypocotyls. *CtFAD2-5* and *-8* appear to be root-specific and *CtFAD2-10* is mostly expressed in flower tissues, with relatively low levels detected in other tissues.

### Expression of safflower *CtFAD2* genes in *Saccharomyces cerevisiae*

**Figure 4:**
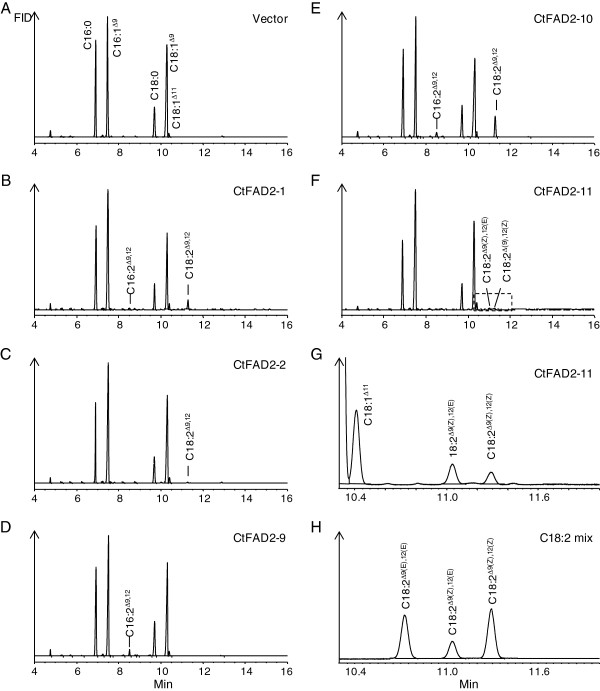
**Fatty acid analysis of yeast expressing empty vector (A), *CtFAD2-1 *(B), *CtFAD2-2 *(C), *CtFAD2-9 *(D), *CtFAD2-10 *(E) and *CtFAD2-11 *(F).** The peaks representing the products by expressing *CtFAD2-11*, including C18:2^Δ9(Z),12(E)^ and C18:2^Δ9(Z),12(Z)^, are highlighted (**G**). FAMEs standard of a mixture of three C18:2 isomers including C18:2^Δ9(E),12(E)^, C18:2^Δ9(Z),12(E)^, C18:2^Δ9(Z),12(Z)^ (**H**).

Expression in yeast (*Saccharomyces cerevisiae)* cells has been successfully used for studying the functional properties of several plant FAD2 Δ12 oleate fatty acid desaturases [19,48,49] because it has a simple fatty acid profile, contains ample oleic acid that can serve as precursor for FAD2 enzymes, and lacks an endogenous FAD2 activity. As shown in Figure 4, polyunsaturated fatty acids including C16:2 and C18:2 were not produced in the control yeast cells transformed with the empty pYES2 vector. By reference to the standard gas chromatogram of C16:2 and C18:2 fatty acid methyl esters (FAMEs) having retention time of 8.513 min and 11.293 min, respectively, C18:2 was found to be present in the yeast lines expressing *CtFAD2-1* (Figure 4B), *CtFAD2-2* (Figure 4C), *CtFAD2-10* (Figure 4E), and C16:2 was seen in yeast lines expressing *CtFAD2-9* (Figure 4D) and *CtFAD2-10* (Figure 4E). This result indicated that CtFAD2-1, CtFAD2-2 and CtFAD2-10 are Δ12 oleate desaturases that convert oleic acid to linoleic acid. It appears that CtFAD2-9 is a Δ12 palmitoleate desaturase that prefers C16:1 to C18:1 substrate, leading to specific production of C16:2. As shown in Figure 4F and Figure 4G, the expression of *CtFAD2-11* resulted in two minor, yet distinct peaks, at retention time of 10.642 min and 11.293 min, respectively. The latter peak is corresponding to FAME of linoleic acid (18:2^Δ9(Z),12(Z)^), and the former peak was identified as FAME of its Δ12 trans isomer (C18:2^Δ9(Z),12(E)^) by GC-MS of their pyrrolidide adducts, and 2,4-dimethyloxazoline (DMOX) modification (Additional file 1: Figure S2). The fatty acid composition of yeast cells expressing *CtFAD2-1, -2, -9, -10* and *-11* is shown in Table 3. As shown in the Table 3, the expression of *CtFAD2-9* produced 1.6% C16:2, without any C18:2. No new peaks were detected in yeast cells expressing *CtFAD2-3*, -*4*, -*5*, -*6*, -*7*, and -*8*. 

**Table 3:** **Fatty acid composition of yeast cells expressing selected *CtFAD2 *genes**

	**vector**	***CtFAD2-1***	***CtFAD2-2***	***CtFAD2-9***	***CtFAD2-10***	***CtFAD2-11***
C14:0	1.3±0.15	1.2±0.02	1.2±0.06	1.1±0.06	1.0±0.01	0.6±0.01
C14:1	0.3±0.06	0.3±0.01	0.3±0.01	0.2±0.01	0.3±0.02	0.2±0.00
C16:0	23.6±1.62	23.0±0.04	22.0±0.46	21.3±0.59	22.3±0.03	18.4±0.34
C16:1	36.0±1.77	37.2±0.16	36.6±0.07	34.3±0.54	34.8±0.21	37.3±0.16
C16:2	0.0	0.3±0.02	0.0	1.6±0.09	1.2±0.03	0.0
C18:0	7.7±0.74	7.4±0.12	7.4±0.07	8.9±0.13	8.1±0.09	7.5±0.07
C18:1	29.6±0.99	26.4±0.24	31.0±0.51	31.3±0.25	25.4±0.07	33.3±0.26
C18:1^Δ11^	1.4±0.20	1.6±0.02	1.5±0.04	1.3±0.08	1.3±0.01	1.9±0.01
C18:2	0.0	2.8±0.14	0.1±0.11	0.0	5.5±0.09	0.3±0.02
C18:2^Δ9(Z),12(E)^	0.0	0.0	0.0	0.0	0.0	0.5±0.03
n=3						

To examine whether any CtFAD2 have fatty acid hydroxylase activity, FAMEs prepared from the yeast cells expressing each of the 11 *CtFAD2* genes were reacted with a silyating reagent that can convert hydroxyl residues into TMS-ether derivatives from which the mass spectrum can be examined. However, none of hydroxyl derivatives of common fatty acids, such as ricinoleic acid synthesised from oleic acid, was detected in any of the yeast cell lines expressing *CtFAD2* genes, indicating the absence of fatty acid hydroxylase activity in the CtFAD2 enzymes. Further tests for potential epoxygenase and acetylenase activities that use linoleic acid as substrate have also been carried out in these transgenic yeast cell lines, by supplementing free linoleic acid in the yeast culture upon the induction by galactose. No novel peaks representing epoxy or acetylenic fatty acids were detected (data not presented).

### Transient expression in *N. benthamiana* leaves

**Figure 5:**
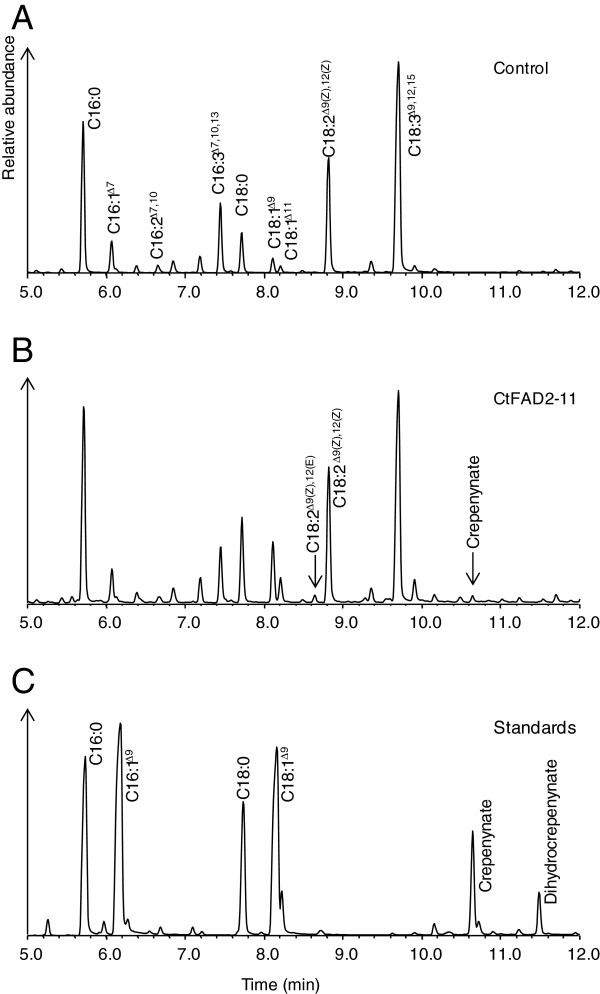
**Fatty acid analysis of *N. benthamiana *leaves transiently expressing *CtFAD2-11.*** (**A**) negative control expressing *35S:P19* alone. (**B**) co-expression of *35S:P19* and *35S:CtFAD2-11*, showing two novel peaks representing the products of CtFAD2-11, C18:2^Δ9(Z),12(E)^ and crepenynic acid. (**C**) fatty acid standard, including the crepenynic acid.

The function of CtFAD2-11 was initially assessed by expression in *S. cerevisiae*, and two novel fatty acids were identified by GC-MS as 18:2^Δ9(Z),12(Z)^ and 18:2^Δ9(Z),12(E)^ respectively. Consistent with the results obtained from yeast, constitutive expression of *CtFAD2-11* driven by 35S CaMV promoter in *N. benthamiana* leaves, co-expressed with *35S:P19* yielded a novel product that is not present in *N. benthamiana* leaf when infiltrated with *35S:P19* alone (Figure 5A). The methyl ester of this new product displayed a GC retention time (8.642 Min) that was identical to that of a methyl 18:2^Δ9(Z),12(E)^ (Figure 5B). The novel 18:2^Δ9(Z),12(E)^ accounted for 0.4% of the fatty acids of leaves transiently expressing *CtFAD2-11* (Table 4). In addition, another new peak that was not observed in the yeast cultures was detected at retention time of 10.642 min (Figure 5B). The total ion chromatogram and mass spectrum of this new fatty acid were consistent with that of crepenynic acid (9-octadecen-12-ynoic acid) (Additional file 1: Figure S3), demonstrating that the CtFAD2-11 polypeptide had Δ12-acetylenase activity as suggested by its close sequence alignments with known Δ12 fatty acid acetylenases. As shown in Table 4, crepenynic acid accounts for 0.5% of total fatty acids in *N. benthamiana* leaves expressing *CtFAD2-11*.

**Table 4:** **Fatty acid composition of *N. benthamiana *leaves transiently expressing *CtFAD2-11***

	**Control**	***CtFAD2*-*11***
C16:0	17.4±0.48	23.7±2.57
C16:1	0.3±0.22	0.3±0.05
C16:2	0.8±0.12	0.6±0.09
C16:3	7.2±0.15	5.5±0.81
C18:0	3.3±0.33	5.3±0.72
C18:1^Δ9^	1.0±0.09	3.8±0.30
C18:1^Δ11^	0.5±0.33	1.2±0.36
C18:2^Δ9(Z),12(E)^	0.0	0.4±0.07
C18:2	12.0±0.65	11.6±0.84
C18:3	56.8±0.19	45.8±4.01
C20:0	0.5±0.10	1.0±0.19
C20:1	0.2±0.15	0.4±0.04
C18:2^Ac^	0.0	0.5±0.06
n=3		

It is notable that the expression of *CtFAD2-11* transiently in the *N. benthamiana* cells resulted in a significant increase of saturated and monounsaturated fatty acids at the expense of polyunsaturated fatty acids including linoleic acid and α-linolenic acid (ALA), relative to the untransformed control (Table 4). Overall, the results from the yeast and *N. benthamiana* expression experiments indicated that CtFAD2-11 functioned primarily as a Δ12 oleate desaturase lacking stereo-specificity, producing both linoleic acid and its trans-Δ12 isomer. In addition, it could also further desaturate the Δ12 double bond of linoleic acid to form the acetylenic bond of crepenynic acid.

The other 10 *CtFAD2* genes have also been expressed in *N. benthamiana* leaves, but we did not observe production of any additional novel fatty acids compared to the controls (data not presented).

## Discussion

We report here the cloning of 11 distinct safflower *CtFAD2* genes, which does not include at least three more partial severely truncated EST sequences that are also present in the CPG safflower EST database. Furthermore, due to the current lack of safflower genomics information it remains unclear whether there are additional unexpressed FAD2 members. Taken together, this is to our knowledge the largest *FAD2* gene family in any species examined to date. In spite of their high conservation of putative amino acid sequences, individual *CtFAD2* genes have clear distinguishing features, especially with respect to their N- and C-terminal ends, 5^′^ and 3^′^ UTR regions, and the size, location and sequence of an intron located at the 5^′^ UTR.

*FAD2* is among the best-studied plant fatty acid desaturase gene families, in terms of both molecular and biochemical investigations. Although only a single representative was identified in Arabidopsis [11], *FAD2* appears to exist as complex gene families in most other plant genomes studied so far. Two distinct *FAD2* genes have been described in soybean [29], flax [50,51] and olive [49]; three genes in sunflower [52] and *Camelina sativa*[53]; and four genes in cotton [31-34]. In the amphitetraploid *Brassica napus*, 4-6 different *FAD2* genes have been identified in each diploid sub-genome [30].

Although comparable studies are lacking, it seems that safflower is unusual with respect to *FAD2* gene family evolution. Safflower is a self-pollinating diploid plant species that is most closely related to a wild diploid species *C. palaestinus* and extensive genome duplication or re-arrangement has not previously been reported in safflower [54]. In the absence of genomic data, it is not known whether the significant amplification in gene copy number observed for FAD2 also occurs for other gene families in safflower. However, our own limited observations of other safflower lipid biosynthesis genes do not show evidence for such expansive gene family size (unpublished data).

The complex FAD2 family cannot be attributed to alternative splicing as *Fad2* genes do not contain introns in the coding region sequence. Instead, gene duplication is more likely responsible for creating the *FAD2* family complexity in safflower. Topology of the gene tree show that gene duplications have probably occurred at several hierarchical levels, i.e. at various times during evolution. For example, the *CtFAD2-3, -4, -5* are more closely related to each other than they are to other safflower *FAD2* sequences, indicating that more recent gene duplications are responsible for the emergence of this clade. It is not known how many *FAD2* genes were generated after the speciation of safflower, in other words, are unique to the *C. tinctorius* species. Analysis of *FAD2* genes from taxonomically closely related plant species, such as those from the genus *Centaurea* within the Asteraceae family, could perhaps provide a better picture of exactly when the duplication events occurred.

Genetic redundancy is a prime feature of plant genomes, providing the opportunity for additional divergent functions to evolve while retaining the original function of vital genes. To persist over long periods of evolutionary time, new gene duplications must be positively selected for, otherwise they can degenerate to pseudogenes by accumulating nonsense mutations, frame shifts and even insertion/deletion events. Once becoming a pseudogene, the likely fate is to continue to accumulate mutations at the maximum rate and become increasingly divergent. The fact that all the 11 *CtFAD2* genes are expressed and translated is evidence that at least these duplicated *FAD2* genes are positively selected in safflower. As the current research focused on the expressed *FAD2* genes, it is plausible that other duplicated *FAD2* genes might have lost expression and functionality through gene pseudogenization or gene deletion.

The production of linoleic acid through the Δ12 desaturation catalyzed by FAD2 is central to the functionality of biological membrane systems, cellular signalling, thermal adaptation, and energy storage [55]. When plants are subject to adverse environmental conditions, a wide range of cellular response occurs, including the adjustments of unsaturation levels of membrane fatty acids. Plant membrane integrity and function, determined by structure and fluidity, are significantly affected by lipid composition, particularly the degree of fatty acid desaturation [23]. Abiotic environmental stresses such as cold, heat, drought and salt are able to induce changes in fatty acid composition. Expression of *FAD2* is also regulated by adverse environmental factors, suggesting the possible involvement of *FAD2* in plant response to abiotic stress [24]. During recent years a number of *FAD2*-divergent genes have been identified in other members of the Asteraceae family that safflower belongs to, such as marigold (*Calendula officinalis*), *Crepis alpina* and sunflower (*Helianthus annuus*) [15,39,56] and have been associated with synthesis of divergent fatty acid structures that may play roles in resistance to biotic stresses. For example, the epoxygenated fatty acids were found to be potent inhibitors for the germination and enlargement of spores of rice blast fungus [57]. Other unusual fatty acids, acetylenic fatty acids, are involved in the biosynthesis of polyacetylenes that act as phytoalexins in numerous Asteraceae species including safflower and therefore play a significant role in plant disease resistance [58-60].

It is intriguing how the divergent *FAD2* gene family members with similar fundamental properties carry out specific functions. The intrinsic characteristics of various *FAD2* genes, as exemplified by some conserved motifs, are likely determining their distinct expression patterns and abilities to respond to environmental stimuli, and diversified functionalities. It has been demonstrated through site directed mutagenesis that very few amino acid changes are required to change the enzymatic function of a *FAD2* gene. For instance, as few as four amino acid changes in a FAD2 fatty acid desaturase were required in order to obtain hydroxylase activity, and conversely, substitution of six amino acids could convert a fatty acid hydroxylase into a fatty acid desaturase [21]. The Δ12 acetylenase from *Crepis alpina* deviates from Δ12 desaturase in 29 positions [15], but it has not been determined how many of these changes are required to facilitate the functionality alteration. It is suggested that a switch from desaturase to acetylenase might involve more extensive changes in sequence than that required to interchange between a fatty acid desaturase and a fatty acid hydroxylase. The origins of specificity leading to acetylenases and desaturases are not currently evident from comparisons at the primary sequence level, and residues promoting acetylenase activity have yet to be located. Interestingly, a Gly replaces Ala at the amino acid immediate preceding the first His box in all the known acetylenases, in contrast to a typical fatty acid desaturase or hydroxylase. It is suggested that this residue may facilitate acetylenase chemistry by alleviating steric hindrance by providing substrate pocket spatial flexibility to permit the conversion of a “kinked” substrate (*cis*-ene) into a straight chain product (acetylene) [61].

We have identified that, although a large functional *FAD2* gene family exists in safflower, only *FAD2-1* has exclusively high expression in developing safflower seeds. It appears that this gene plays the major role in producing the very high levels of linoleic acid present in seed storage oils of the wild-type (high-linoleic) safflower. FAD2-2, the presumed house-keeping microsomal Δ12 oleate desaturase has a generally constitutive expression throughout the plant. It is interesting to note that in this study we have found that *CtFAD2-2* has significantly higher expressioin in cotyledonary tissues of a young seedling, compared to all other safflower tissues examined. Although likely contributing to the overall oleate desaturase activity in safflower vegetative tissues (where Fad6 also plays a significant role), CtFAD2-2 presumably plays only a secondary role in linoleic acid production in the seed oil.

The expression of *CtFAD2-9* in yeast led to the specific synthesis of palmitolinoleic acid (C16:2^Δ9,12^), presumably produced by the desaturation of palmitoleic acid. It is well established that the initial desaturation of a C16 saturated fatty acid, palmitic acid occurs in the plastids by the action of the stearoyl-ACP Δ9-desaturase to form palmitoleic acid that could be exported to the cytosol and incorporated into phospholipids for further desaturation by a FAD2 oleate Δ12-desaturase [12]. To our knowledge, the safflower CtFAD2-9 is the first described FAD2 enzyme specific for the production of palmitolinoleic acid. Palmitolinoleic acid does not normally accumulate in abundance in plant tissues as it is quickly converted to hexadecatrienoic acid (C16:3) by abundant Δ15-desaturase in vegetative tissues, and in seed tissue there is limited availability of C16:1 substrate. In olive leaves and mesocarp tissues, increased accumulation of palmitolinoleic acid was observed in respond to wounding [62]. It was suggested that the increase of palmitolinoleic acid in wounded olive mesocarp is a result of enhanced demand for substrates for the synthesis in the chloroplast of hexadecatrienoic acid that could be used as precursors *via* the lipoxygenase pathway of signal molecules involved in plant defence and wounding [63]. However, the involvement of C16 polyunsaturated fatty acids in the oxylipin biosynthesis is less documented compared to its C18 counterparts. It remains to be revealed what the specific evolutionary drive is for CtFAD2-9 that specifically produces palmitolinoleic acid in safflower.

Among the 11 safflower *CtFAD2* genes, *CtFAD2-10* is the first characterised *FAD2* gene with preferential expression in safflower heads prior to seed set. However this is similar to the situation in cotton where of the four different genes encoding FAD2 isolated one member, *ghFAD2-1,* was specifically expressed in both developing flower buds and seeds [33]. It is known that jasmonic acid derived from ALA plays a significant role as a chemical signal controlling stamen and pollen development, especially in the final stages of pollen development and anther dehiscence in Arabidopsis [28]. As the precursor of ALA, therefore, the enhanced production of linoleic acid by FAD2 may play an essential role in flower development. It is likely that the retention of *CtFAD2-1* as a strong Δ12-desaturase for synthesis of seed storage lipids has freed up the duplicated *CtFAD2-10* for diversification into a specialised flower-specific form. Evolutionary modifications of the *CtFAD2-10* gene that favoured flower expression at the expense of seed expression would be unlikely to have any negative fitness consequences given the retention of *CtFAD2-1* seed expression and the relatively inconsequential impact that moderate variations in oleic-linoleic ratios in seed lipids would be expected to have on fitness.

We demonstrated that the CtFAD2-11 Δ12 acetylenase is a tri-functional enzyme that could desaturate both oleate and linoleate. It produces a mixture of Δ12(Z) and (E) isomers of C18:2 from oleate in yeast cells. In plant cells, CtFAD2-11 could also produce crepenynic acid (C18:2^Δ9(Z)^,-octadecen-12-ynoate) from linoleate, as demonstrated by transient expression in a *N. benthamiana* leaf. Such a multi-functionality has previously been reported in *Crepis alpina* where CREP1 could produce both *cis* and *trans* isomers of C18:2 fatty acid, in addition to producing crepenynic acid in transgenic Arabidopsis [22]. When expressed in yeast, CtFAD2-11 produces a higher level of C18:2^Δ9(Z),12(E)^ than linoleic acid, consistent with the expression of CREP1 in Arabidopsis seeds [22]. C18:2^Δ9(Z),12(E)^ has been identified as an apparent metabolic dead end as it cannot be converted further into crepenynic acid. The *cis* and *trans* FAD2-mediated Δ12 oleate desaturation likely have very similar catalytic mechanisms but differ in substrate binding properties. Compared to a normal FAD2 oleate *cis*-Δ12 desaturase, CtFAD2-11 might have an altered substrate interaction geometry at the active site. A less restrictive binding pocket could allow a small rotation of the substrate and permit the formation of an E/Z mixture. It was previously demonstrated that a C18:2^Δ9(Z),12(Z)^ is produced by abstraction of the pro-R hydrogens at C12 and C13, whereas and C18:2^Δ9(Z),12(E)^ is produced by scission of the (12R,13S) hydrogens [22].

It appears that the Δ12 *trans*-double bond is a co-product of acetylenic fatty acid production by a specialised FAD2 enzyme in both safflower and Crepis. The *trans* desaturation of oleate to form C18:2^Δ9(Z),12(E)^ has also been shown by another functionally divergent FAD2 enzyme, the Δ12 fatty acid conjugase from tung (*Aleurites fordii*), which displayed *trans*-Δ12 oleic acid desaturase activity, in addition to its conjugase activity [19]. The production of a *trans* double bond, accompanied by the production of other unusual fatty acids, such as calendic acid (C18:3^8(E),10(E),12(Z)^) in Calendula, and dimorphecolic acid (9OH-18:2^Δ10(E),12(E)^) in *Dimorphotheca sinuate* by divergent FAD2 fatty acid modifying enzymes has also been reported [20,45,64].

In the transient expression system of *N. benthamiana* leaves, we have observed that the expression of *CtFAD2-11* resulted in an increase in the relative proportions of saturated and monoenoic fatty acids at the expense of polyunsaturated fatty acids. This is consistent with what has previously been observed in the expression of acetylenases and epoxygenases in Arabidopsis and cotton [65,66]. The production of vernolic acid, by modest seed-specific expression of the *C. palaestina* Δ12-epoxygenase (*Cpal2*) gene in Arabidopsis and cotton seeds led to enhanced oleic acid accumulation at the expense of linoleic acid compared to the wild type seeds. The subsequent co-expression of *C. palaestina* Δ12 desaturase (*Cpdes*) with *Cpal2* was shown to restore the normal oleate desaturation and therefore further raised the vernolic acid level. This highlights that substrate availability is one factor controlling diverged FAD2 desaturase activity. Potential alternative mechanisms for the reduced levels of linoleic acid in *N. benthamiana* leaves expressing the *CtFAD2-11* gene could include a degree of homology-based post-transcriptional silencing of Δ12-desaturase members of the *FAD2* gene family that are operational in the leaf, or the possible formation of potentially ineffective heterodimers between CtFAD2-11 and the FAD2 Δ12-desaturase proteins [67].

Based on sequence alignments CtFAD2-11 is most closely related to the sunflower vFAD2 that is characterized as a fungal elicitor-inducible Δ12 fatty acid acetylenase and could produce both crepenynate and (14Z)-dehydrocrepenynate when expressed in developing soybean somatic embryos [39]. Divergent *FAD2* genes with the same catalytic functionality as the sunflower *vFAD2* have also been isolated from numerous plant species, including the *EIl12* from parsley (*Petroselinum crispum* L*.*), *C. officinalis* and English ivy (*Hedera helix* L.) [39]. Although sharing higher sequence homology to vFAD2 than *C. alpina* CREP1, when expressed in *N. benthamiana* CtFAD2-11 was shown to have the same functionality as CREP1, it did not have the additional synthesis of (14Z)-dehydrocrepenynic acid typical of the *vFAD2*-like genes.

Accumulation of acetylenic fatty acids or any other unusual fatty acids that would be synthesized by divergent forms of FAD2 enzymes was not observed in various tissues of safflower, consistent with previous findings with *C. alpina* and *H. annuus* flower tissue even though gene expression was detected [39,68]. Identifying the function and control mechanisms of such cryptic expression of unusual fatty acids is a poorly explored aspect of fatty acid metabolism. It is likely that acetylenic fatty acids are synthesized in low amounts and are rapidly metabolized for the formation of secondary bioactive molecules. Crepenynic and dehydrocrepenynic acids are believed to be intermediates in the biosynthetic pathway of biologically active polyacetylenic compounds that are known to occur in safflower as well as some other members of the Asteraceae, Apiaceae, and Araliaceae families [59,60,69,70]. However, many of the genes necessary for the formation of the putative antifungal polyacetylenes need to be discovered and the *in vivo* functions of functionally divergent FAD2(s) in the polyacetylene pathway remain to be established.

In addition to the above mentioned safflower *FAD2* genes, another six *CtFAD2* genes, from *CtFAD2-3* through to *CtFAD2-8*, are highly expressed in rapidly expanding tissues such as cotyledons, hypocotyls, and roots in the young safflower seedlings. It is not apparent why multiple *FAD2* genes would be expressed in young seedling tissues in safflower, although enhanced FAD2 expression and increased linoleic acid production was previously observed in sunflower cotyledons immediately upon germination [52,71]. However, these *CtFAD2* genes are possibly more likely to be performing reactions other than oleate desaturases, considering that their primary sequence features are more closely associated with functionally divergent FAD2 homologs and they were unable to synthesise linoleic acid when expressed in yeast.

## Conclusions

The identification and initial characterization of the 11 full length *FAD2* cDNAs and their corresponding structural genes provides an insight into the principal determinants of synthesis of linoleic acid in safflower seed oil. It also presents an unprecedented opportunity to fully understand the fundamental functions and diversity of FAD2 proteins and their differing fatty acid modifications within a single plant species. Characterization of the biochemical properties of each *FAD2* gene will lend further insight into their potential roles. Identifying the specific structural attributes of the diverged FAD2 enzymes that control their regio- and chemo- selectivity would be greatly accelerated by availability of a structure-function characterization of the microsomal fatty acid desaturases, an elusive goal that would have a wide ranging impact on lipid metabolic research. Defining the phenotype of transgenic plants with RNAi down regulation of each *FAD2* gene will contribute to the clarification of the pathways attributed by FAD2 members. Ultimately, the use of divergent FAD2 enzymes to produce novel fatty acids, such as crepenynic acid, in genetically engineered crop plants may not only provide opportunities for protection from disease or pests to the plant but also allow for the synthesis of economically valuable oleochemical products.

## Methods

### Plant materials and growth conditions

Seed of wild type (high-linoleic) safflower (*Carthamus tinctorius* L.) was sourced from a commercial birdseed supplier in Australia and was designated as genotype SU. SU safflower plants were grown from seed in the glasshouse in a perlite and sandy loam potting mix under a day/night cycle of 16 hrs (25°C)/8 hrs (22°C). Plant tissues including leaves, roots, cotyledons and hypocotyls were sampled from young germinating safflower seedlings. Flowering heads were obtained at the first day of flowering and developing embryos were harvested at three stages representing early, middle and late seed development. Samples were immediately chilled in liquid nitrogen and stored at -80°C until DNA and RNA extraction.

### Total RNA extraction and cDNA synthesis

Total RNA isolation was performed from 100 mg of frozen safflower tissues using RNeasy® Plant total RNA kit (Qiagen, Hilden, Germany) as described by manufacturer’s protocol. RNA concentration was determined by NanoDrop™ spectrophotometer ND1000 (Thermo Fisher Scientific, Victoria, Australia) and concentrations were equalized before analysis. Quality and relative quantities were also visualized by standard RNA formaldehyde agarose gel (1%, w/v). Total RNA was then treated with RQ1 RNase-free DNase (Qiagen, Hilden, Germany) to remove contaminating genomic DNA. First-strand cDNA was synthesized from 400 ng of DNA-free total RNA using the SuperScript III First-Strand Synthesis System (Qiagen, Hilden, Germany) with oligo(dT)_20_ primer, following the manufacturer’s instructions.

### Construction and screening of cDNA library derived from safflower developing embryos

To isolate *FAD2* cDNAs a lambda cDNA library was constructed using safflower developing embryos. A mixture of immature embryos of different developmental stages were harvested and ground to powder in liquid nitrogen and RNA extraction was carried out using TRIzol following the manufacturer’s instruction (Invitrogen, Carlsbad, CA, USA). Poly(A)-containing RNA was isolated using Qiagen mRNA purification kit (Qiagen, Hilden, Germany). First strand oligo(dT)-primed cDNA was synthesised and converted to double stranded using Stratagene cDNA synthesis kit, according to the manufacturer’s instructions (Stratagen, La Jolla, CA, USA). The blunt-ended cDNA was ligated with *Eco*RI adaptors, phosphorylated, and size fractionated by gel-filtration in a Chroma spin+TE-400 column (Clontech, CA, USA). The recombinants were propagated in the *E. coli* strain XL-1 Blue MRF’ using a Stratagene Predigested λZAP II/*Eco*RI/CIAP cloning kit (Invitrogen, Carlsbad, CA, USA).

To identify the *FAD2* cDNAs, the cDNA library was screened using a DNA fragment corresponding to the coding region of Arabidopsis *FAD2* following the protocol previously described [31]. Positive plaques were purified through two successive rounds of screening and the purified phagemids containing putative *FAD2* cDNAs were excised as outlined in the Stratagene λZAPII cDNA Synthesis Kit instruction manual (Invitrogen, Carlsbad, CA, USA). Sequence analysis of the *FAD2* sequences were identified by the NCBI Blast program (http://www.ncbi.nlm.nih.gov/BLAST/). The open reading frame was predicted by using Vector NTI.

### Isolation of the full length and the open reading frame (ORF) of *FAD2* genes expressed in non-seed tissues

We have also interrogated the Composite Genome Project (CGP) expressed sequence tag (EST) database of safflower (http://cgpdb.ucdavis.edu/cgpdb2.), which was screened by “Blast” for similarity with Arabidopsis *FAD2* sequence (AT3G12120). Putative safflower *FAD2* ESTs were compared and the longest EST clones of the strongest hits from different contigs were selected for extension into full length by performing 3^′^ Rapid Amplification of cDNA Ends (RACE) using Bioline one step RT-PCR kit following Manufacturers’ instructions (Bioline, London, UK). A gene-specific primer (GSP) was used for each of the selected ESTs, in combination with a poly(dT) primer with a *Not*I site at its 3^′^ end. A second round of PCR was performed using a nested GSP in combination with the poly(dT) primer. GSPs for 3^′^ RACE were listed in Additional file 1: Table S1. The one-step reverse transcriptase PCR reactions were performed using 200 ng of RNA as templates starting with an initial reverse transcription reaction at 50°C for 30 min, followed by 95°C for 3 min, and 40 cycles at 95°C for 30 s, 55°C for 30 s, and 72°C for 1 min, and a final extension at 72°C for 10 mins. Cloning of the 5^′^ end of the *CtFAD2-6* cDNA was performed with 5^′^ RACE System Kit (Invitrogen, Carlsbad, CA, USA) following manufacturers’ instruction. Only the *CtFAD2-6* mRNA was reverse transcribed to cDNA using a gene-specific primer GSP1, 5^′^- ACCTAACGACAGTCATGAACAAG -3^′^. A gene-specific primer GSP2, 5^′^- GTGAGGAAAGCGGAGTGGACAAC -3^′^ was used in the first PCR amplification. A hot start at 95°C for 4 min before adding the polymerase, 33 cycles of denaturation at 94°C for 45 s, annealing at 55°C for 1 min and extension at 72°C for 2 min. The amplification products were fractionated on 1% agarose gel from which the DNA fragments with expected sizes were purified and subcloned into the vector pGEM-Teasy® (Promega, Madison, WI, USA) and identity confirmed by DNA sequencing using an ABI 373 sequencer.

The ORFs of *FAD2* genes were amplified using the same One-step RT-PCR kit (Bioline, London, UK) on total RNAs derived from several safflower plant tissues and PfuUltra II Fusion HS DNA Polymerase (Stratagen, La Jolla, CA, USA). The primers (Additional file 1: Table S2) used to amplify the ORFs were designed based on the DNA sequences located in the 5^′^ and 3^′^ UTR of each gene. The amplified PCR products were cloned to vector pGEM-Teasy® (Promega, Madison, WI, USA), and verified by DNA sequencing. The cloned amplification products were addressed with the gene name *FAD2* and a suffix representing the first letter of the genus (C for *Carthamus*) and species (t for *tinctorius*).

### DNA isolation and Southern blot analysis

The genomic DNA of safflower genotype SU was isolated from fully expanded leaves using CTAB buffer following the method described by Paterson *et al.* (1993). Further purification was carried out using CsCl gradient as previously described [31]. About 10 μg safflower genomic DNA was digested with seven different restriction enzymes, namely *Acc*I, *Bgl*II, *Bam*HI, *Eco*RI, *Eco*RV, *Hin*dIII, *Xba*I and *Xho*I.

Genomic DNA digested with each restriction enzyme was electrophoresed through 1% agarose gel. The gel was soaked in 0.5 M NaOH, 1.5 M NaCl for 30 min and blotted onto a Hybond-N^+^ nylon membrane (Amersham, UK). The filters were probed with α-P^32^ dCTP-labeled safflower *FAD2* DNA fragment. The hybridization was performed in 6x SSPE, 10% Denhardt’s solution, 0.5% SDS, 100 ug/mL denatured salmon sperm DNA overnight at 65°C. After a brief wash in 2x SSC/0.1% SDS at 50°C, the filter was washed three times in 0.2 x SSC/0.1% SDS at 50°C for 20 min each prior to autoradiography.

### The isolation of 5^′^ UTR intron of *CtFAD2* genes

In order to isolate the DNA sequences of an intron that is situated at the 5^′^ UTR of *CtFAD2* genes, the typical splice site (AG:GT) was predicted in the 5^′^ UTR of each *CtFAD2* cDNA sequence, and PCR primers were designed based on the flanking sequences of predicted splice site. The primers are listed in Additional file 1: Table S3. Genomic DNA isolated from safflower genotype SU was used as the template. The amplification was accomplished in 50 μL reactions with 100 ng of genomic DNA, 20 pmol of each primer, and a Hotstar (Qiagen, Hilden, Germany) supplied by the manufacturer. PCR temperature cycling was performed as follows: 94°C for 15 min for one cycle, 94°C for 30 s, 55°C for 1 min, 72°C for 2 min for 35 cycles; 72°C for 10 min using the Kyratec supercycler SC200 (Kyratec, Queensland, Australia). The PCR products were cloned into pGEM-T Easy® (Promega, Madison, WI, USA) following manufacturer’s instructions, prior to DNA sequence determination.

### Real-Time quantitative PCR

Gene expression analysis was performed by RT-qPCR using BIORAD CFX96™ Real-time PCR detection system and iQTM SYBR® Green Supermix (BioRad, Hercules, CA, USA). Primers with Tm (melting temperature) of about 65°C and 19-23 bp in length were designed for gene-specific amplification of a product about 100-200 bp fragments (Additional file 1: Table S4). PCR reactions were carried out in 96-well plates. All reactions were performed in triplicates. Reaction mix (10 uL per well) contained 1 x iQTM SYBR® Green Supermix (BioRad, Hercules, CA, USA), 5 μM forward and reverse primers, and 400 ng of cDNA. The thermal cycling conditions were 95°C for 3 min, followed by 40 cycles of 95°C for 10 s, 60°C for 30 s and 68°C 30 s. The specificity of the PCR amplification was monitored by melting curve analysis following the final step of the PCR from 60°C through 95°C at 0.1°C/sec. Additionally, PCR products were also checked for purity by agarose gel electrophoresis and confirmed by sequencing. A constitutively expressed β-ketoacyl-acyl-carrier protein (ACP) synthase II (*KASII*) gene was used as an endogenous reference. KASII is responsible for the elongation of C16:0-ACP to C18:0-ACP in the *de novo* fatty acid biosynthesis in plants. The suitability of safflower *KASII* gene as an internal reference gene was validated by its high expression stability in various tissues and developmental stages (unpublished data). The data were calibrated relative to the corresponding gene expression level following the 2^-△△Ct^ method for relative quantification [72]. The data were presented as means ± SD of three reactions performed on independent 96-well plates.

### Expression of *CtFAD2* genes in *Saccharomyces cerevisiae*

The DNA fragment containing the entire open reading frames of safflower *CtFAD2* cDNAs were excised from pGEM-Teasy vector as an *Eco*RI fragment and re-ligated to the corresponding site of the vector pENTR11 (Invitrogen, Carlsbad, CA, USA) and then cloned to the destination expression vector pYES2-DEST52 using the Gateway® Cloning recombination technology (Stratagen, La Jolla, CA, USA) that has GAL1 promoter for inducible gene expression. The gene sequences in these plasmids were each verified by DNA sequence determination. The resulting plasmids and the pYES2-DEST52 vector lacking cDNA insert were introduced into bakers’ yeast (*Saccharomyces cerevisiae*) YPH499 cells by lithium acetate-mediated transformation. Expression of these *CtFAD2* genes in yeast cells with or without exogenous fatty acid substrate feeding was essentially as previously described [73]. Each experiment was carried out in triplicate.

### Transient expression of *CtFAD2* in *Nicotiana benthamiana* leaves

Each of the *CtFAD2* ORFs was cloned in sense orientation into a modified pORE04 binary vector between the double CaMV-35S promoter and an *Agrobacterium tumefaciens* NOS terminator containing polyadenylation signal sequence [74]. A vector constitutively expressing the viral suppressor protein, P19, was obtained from Dr Peter Waterhouse’s lab. Previous research indicated that the expression of transgenes could be significantly enhanced by the co-expression of P19 to prevent host transgene silencing in *N. benthamiana* leaf-based transient assay [16,75,76]. *A. tumefaciens* strain AGL1 harbouring the *35S:CtFAD2* was grown at 28°C with shaking in LB broth supplemented with the 50 mg/L kanamycin for two days prior to pelleting by centrifugation and resuspension in 1 mL of infiltration buffer containing 5 mM MES, 5 mM MgSO_4_ and 100 μM acetosyringone and cultured at 28°C with shaking for an additional three hours. The 10x dilution of each culture was then diluted by 10 times with the infiltration buffer and mixed with equal volume of the *35S:P19* culture and infiltrated into the underside of the fully expanded *N. benthamiana* leaves as described by Voinnet *et al. *[16]. Following a period of 5 days further growth at 24°C, the infiltrated leaf regions were excised and immediately subjected to fatty acid analysis.

### Fatty acid analysis

Fatty acid methyl esters (FAME) were prepared by transesterification of the total fatty acids in yeast cells, obtained as cell pellets after centrifugation of cultures [73], or in *N. benthamiana* leaf, by adding 750 μL of 1 N MeOH-HCl (Supelco) at 80°C for minimal 2 hrs, then added 500 μL of 0.9% NaCl. FAMEs were extracted with 300 μL of hexane, and analysed by GC with Agilent 7890A GC on a 30-m BPX70 column essentially as described before [77] except that the ramping program changed to initial temperature at 150°C, holding 1 min, ramping 3°C/min to 210°C, the 50°C/min to 240°C for a final holding 2 min. Confirming double bond positions in the FAME by 2,4-dimethyloxazoline (DMOX) modification and GC-MS analysis were carried out same as previously described [77], except with a Shimadzu GC-MS QP2010 Plus on a 30-m BPX70 column. The column temperature was programmed as an initial temperature at 150°C for 1 min, ramping at 5°C/min to 200°C then 10°C/min to 240°C with holding for 5 min. Mass spectra were acquired and processed with GCMS solution software (Shimadzu, Version 2.61). The free fatty acids and FAME standards were purchased from Sigma-Aldrich (St. Louis, MO, USA).

## Abbreviations

*FAD2*: Microsomal Δ12 fatty acid desaturase; CtFAD2: *Carthamus tinctorius* FAD2; ER: Endoplasmic reticulum; EST: Expressed sequence tag; NCBI: National Center for Biotechnology Information; PCR: Polymerase chain reaction; RT-qPCR: Real time quantitative reverse transcription polymerase chain reaction.

## Competing interests

The authors declared that they have no competing interests.

## Authors’ contributions

SC participated in the experimental design, performed experiments, analysed data, and wrote the manuscript. XRZ participated in the experimental design, performed experiments, CCW, AGG and SPS also participated in the experimental design and writing of manuscript, LL participated in manuscript preparation and supervision of experiments, QL participated in the experimental design, performed experiments, supervised all procedures, analysed data and wrote the manuscript. All authors read and approved the final manuscript.

## Supplementary Material

Additional file 1 **Figure S1.** Sequence alignment of the putative polypeptides derived from the 11 safflower CtFAD2 and orthologous plant FAD2s. atDES, AAM61113.1; lcDES, ACR15954.1; pfOH:DES, AAC32755.1; plOH, ABQ01458.1; coCONJ, AAK26632.1; haACET, ABC59684.1; rhACET, AAO38035.1; dsACET, AAO38036.1; caACET, ABC00769.1; cpEPOX, CAA76156.1; slEPOX, AAR23815.1; dcACET, AAO38033.1; dcDES:OH, AAK30206.1; fvACET,AAO38034.1; hhACET, AAO38031.1; boDES, AAC31698.1; haDES-2, AAL68982.1; haDES-3, AAL68983.1; haDES-1, AAL68981.1; ntDES,AAT72296.2; oeDES, AAW63040; siDES, AAF80560.1; ghDES-1, CAA65744.1; ptDES, XP_002297660.1; rcOH, AAC49010.1; cpDES, AAS19533.1; ghDES-4, AAQ16653.1; ghDES-2, CAA71199.1; jcDES, ADB93805.1; luDES,ACF49507.1. **Figure S2.** Mass spectral identification of DMOX derivatives of C18:2^Δ9(Z),12(E)^ and C18:2^Δ9(Z),12(Z)^ from *S. cerevisae* expressing *safflower CtFAD2-11*. **Figure S3.** Mass spectral identification of DMOX derivatives of crepenynic acid (9-octadecen-12-ynoic acid) from *N. benthamiana* leaves transiently expressing *safflower CtFAD2-11* (A). methyl crepenynate standard. **Table S1.** Oligonucleotide primers used in the 3′RACE of multiple *CtFAD2* genes in safflower **Table S2.** Oligonucleotide primers used for amplification of the entire coding region of *CtFAD2* genes in safflower. **Table S3.** Oligonucleotide primers used for the amplification of 5′UTR intron of *CtFAD2* genes in safflower. Table S4. Oligonucleotide primers used for RT-qPCR in the expression profile study of safflower *CtFAD2* genes. Click here for file
