# Supplementary material for: A large and functionally diverse family of Fad2 genes in safflower (Carthamus tinctorius L.)
Source: BMC Plant Biol. 2013 Jan 7;13:5. doi: 10.1186/1471-2229-13-5 (PMC3554562; doi:10.1186/1471-2229-13-5)
Supplement: Additional file 1 — Figure S1. Sequence alignment of the putative polypeptides derived from the 11 safflower CtFAD2 and orthologous plant FAD2s. atDES, AAM61113.1; lcDES, ACR15954.1; pfOH:DES, AAC32755.1; plOH, ABQ01458.1; coCONJ, AAK26632.1; haACET, ABC59684.1; rhACET, AAO38035.1; dsACET, AAO38036.1; caACET, ABC00769.1; cpEPOX, CAA76156.1; slEPOX, AAR23815.1; dcACET, AAO38033.1; dcDES:OH, AAK30206.1; fvACET,AAO38034.1; hhACET, AAO38031.1; boDES, AAC31698.1; haDES-2, AAL68982.1; haDES-3, AAL68983.1; haDES-1, AAL68981.1; ntDES,AAT72296.2; oeDES, AAW63040; siDES, AAF80560.1; ghDES-1, CAA65744.1; ptDES, XP_002297660.1; rcOH, AAC49010.1; cpDES, AAS19533.1; ghDES-4, AAQ16653.1; ghDES-2, CAA71199.1; jcDES, ADB93805.1; luDES,ACF49507.1. Figure S2. Mass spectral identification of DMOX derivatives of C18:2Δ9(Z),12(E) and C18:2Δ9(Z),12(Z) from S. cerevisae expressing safflower CtFAD2-11. Figure S3. Mass spectral identification of DMOX derivatives of crepenynic acid (9-octadecen-12-ynoic acid) from N. benthamiana leaves transiently expressing safflower CtFAD2-11 (A). methyl crepenynate standard. Table S1. Oligonucleotide primers used in the 3′RACE of multiple CtFAD2 genes in safflower Table S2. Oligonucleotide primers used for amplification of the entire coding region of CtFAD2 genes in safflower. Table S3. Oligonucleotide primers used for the amplification of 5′UTR intron of CtFAD2 genes in safflower. Table S4. Oligonucleotide primers used for RT-qPCR in the expression profile study of safflower CtFAD2 genes. [file 1471-2229-13-5-S1.docx]

Additional files

Additional file 1_ Figure S1. Sequence alignment of the putative polypeptides derived from the 11 safflower CtFAD2 and orthologous plant FAD2s.

atDES, AAM61113.1; lcDES, [ACR15954.1](http://www.ncbi.nlm.nih.gov/protein/237692504?report=genbank&log$=prottop&blast_rank=2&RID=XG6MBGEP01N); pfOH:DES, AAC32755.1; plOH, ABQ01458.1; coCONJ, AAK26632.1; haACET, ABC59684.1;

rhACET, [AAO38035.1](http://www.ncbi.nlm.nih.gov/protein/31322141?report=genbank&log$=prottop&blast_rank=1&RID=XG63PE6X01N); dsACET, AAO38036.1; caACET, ABC00769.1; cpEPOX, CAA76156.1; slEPOX, [AAR23815.1](http://www.ncbi.nlm.nih.gov/protein/38564776?report=genbank&log$=prottop&blast_rank=1&RID=XG789Y1P01N); dcACET, AAO38033.1;

dcDES:OH, [AAK30206.1](http://www.ncbi.nlm.nih.gov/protein/13560785?report=genbank&log$=prottop&blast_rank=2&RID=XG7F0E7K01S); fvACET,[AAO38034.1](http://www.ncbi.nlm.nih.gov/protein/31322139?report=genbank&log$=prottop&blast_rank=1&RID=XG7HV8J901S); hhACET, AAO38031.1; boDES, [AAC31698.1](http://www.ncbi.nlm.nih.gov/protein/3417601?report=genbank&log$=prottop&blast_rank=1&RID=XG7SPNW4016); haDES-2, AAL68982.1; haDES-3, [AAL68983.1](http://www.ncbi.nlm.nih.gov/protein/18418653?report=genbank&log$=prottop&blast_rank=1&RID=XG83WXH601N);

haDES-1, [AAL68981.1](http://www.ncbi.nlm.nih.gov/protein/18418649?report=genbank&log$=prottop&blast_rank=1&RID=XG86TB4V01S); ntDES,[AAT72296.2](http://www.ncbi.nlm.nih.gov/protein/51556906?report=genbank&log$=prottop&blast_rank=1&RID=XG8EYV2U01S); oeDES, AAW63040; siDES, AAF80560.1; ghDES-1, CAA65744.1; ptDES, [XP_002297660.1](http://www.ncbi.nlm.nih.gov/protein/224053006?report=genbank&log$=prottop&blast_rank=1&RID=XG8WEHNV01S); rcOH,

AAC49010.1; cpDES, [AAS19533.1](http://www.ncbi.nlm.nih.gov/protein/42541363?report=genbank&log$=prottop&blast_rank=1&RID=XG90VTUY01S); ghDES-4, AAQ16653.1; ghDES-2, CAA71199.1; jcDES, [ADB93805.1](http://www.ncbi.nlm.nih.gov/protein/284794949?report=genbank&log$=prottop&blast_rank=1&RID=XG955407012); luDES,[ACF49507.1](http://www.ncbi.nlm.nih.gov/protein/194346322?report=genbank&log$=prottop&blast_rank=1&RID=XG994GB501N)

1 135

atDES (1) MGAGGRMP---VPTSSKKSET-DTTKRVPCEKPPFSVGDLKKAIPPHCFKRSIPRSFSYLISDIIIASCFYYVATNYFSLLPQPLS-YLAWPLYWACQGCVLTGIWVIAHECGHHAFSDYQWLDDTVGLIFHSFL

lcDES (1) MGAGGRMP---VPPSSKKTET-EALKRVPCEKPPFTLGDLKKAIPPQCFKRSIPRSFSYLISDIIIASCFYYVATNYFSLLPQSIS-YLAWPLYWACQGCVLTGIWVIAHECGHHAFSDYQWLDDTVGLIFHSFL

pfOH:DES (1) MGAGGRIM---VTPSSKKSET-EALKRGPCEKPPFTVKDLKKAIPQHCFKRSIPRSFSYLLTDITLVSCFYYVATNYFSLLPQPLSTYLAWPLYWVCQGCVLTGIWVIGHECGHHAFSDYQWVDDTVGFIFHSFL

plOH (1) MGAGGRIM---VTPSSKKSKP-EALRRGPGEKPPFTVQDLRKAIPRHCFKRSIPRSFSYLLTDIILASCFYYVATNYFSLLPQPLSTYFAWPLYWVCQGCVLTGVWVLGHECGHQAFSDYQWVDDTVGFIIHTFL

coCONJ (1) MGAGGR-------MSDPSEG-KNILERVPVDPP-FTLSDLKKAIPTHCFERSVIRSSYYVVHDLIVAYVFYYLANTYIPLIPTPLA-YLAWPVYWFCQASILTGLWVIGHECGHHAFSDYQLIDDIVGFVLHSAL

haACET (1) MGAGGR-------MSDPSEG-KNILERVPIDPP-FTLSDLKKAIPAHCFERSVIRSSYYVVHDLIVAYVFYFLANTYIPLLPTPWA-YLAWPVYWFCQASILTGLWVIGHECGHHAYSDYQLIDDIVGFVLHSAL

rhACET (1) -----------------------------------------KAIPPHCFERSVIRSSYYVVHDLIVAYVFYFLANTYIPLLPTPWA-YLAWPVYWFCQASILTGLWVIGHECGHHAFSDYQLIDDLVGFVLHSA

ctFAD2-11 (1) MGAGGR-------MSDSSESSTDILKRVPIDPP-FTLSDLKKAIPARCFERSLIRSSYYVVHDLVVAYVFYYLADTYIPQLPTPLA-YLAWPIYWFCQASICTGLWVIGHECGHHAFSEQQWIGDIVGIVLHSAL

dsACET (1) ----------------------------------------KKAIPPHCFERSLIRSSYYVVHDLIVAYVFYFLANTYIPLLPAPLA-YLAWPVYWFCQASILTGLWVIGHECGHHAFSEQQWIGDTVGFILHSAL

caACET (1) MGGGGR-------GRTSQKP---LMERVSVDPP-FTVSDLKQAIPPHCFKRSVIRSSYYIVHDAIIAYIFYFLADKYIPILPAPLA-YLAWPLYWFCQASILTGLWVIGHECGHHAFSDYQWVDDTVGFILHSFL

cpEPOX (1) MGAGGR-------GRTSEKS---VMERVSVDPVTFSLSELKQAIPPHCFQRSVIRSSYYVVQDLIIAYIFYFLANTYIPTLPTSLA-YLAWPVYWFCQASVLTGLWILGHECGHHAFSNYTWFDDTVGFILHSFL

slEPOX (1) ---MSD-------SYDDRMKDHDMDERAPIDPAPFSLSDLKKAIPAHCFRRSAVWSSCYVVQDLIITFLLYTVANTYIPHLPPPLV-YLAWPVYWFCQSCILTGLWVLGHECGHHAFSEYQWIDNAVGFVLHSAL

ctFAD2-3 (1) MGAGGRMNAEVSG-----SEKPHAFKRVPVSKPPFELSDLKKAVPPHCFKRSLVRSFAALFRDIIIVTALYYLAATIIPVLPKPLT-YVAWPLYWFFQGAYLMGLWVIGHECGHHGFSEYQWLDDTVGFIVHSLI

ctFAD2-4 (1) MGAGGRMDVAAS------SEKPHAFKRVPVSKPPFELSDLKKAVPPHCFKRSLVRSFAALFRDIIIVSALYYLAATVIPVLPKPLT-YVAWPLYWFFQGAYLMGLWVIGHECGHHGFSEYQWLDDTVGFIVHSLI

ctFAD2-7 (1) MGAGGRMKTEEK----------DIMKHVPVAKPPFGISDLKKATPPHCFKRSLIWSFGSFFRDLIVIYSFYYLASTFIPLLPPALS-YVAWPLYWFAQGSILMGFWILGHECGHHAFSEYQWVDDAVGFFIHSVC

ctFAD2-6 (1) MGAGGRMNDATT--------DKDVLKRVPTAKTPFEISDLKKAIPPHCFKRSLTTSFYYLFRDICICYTWYHLGSNYLPLLPKPLA-YIAWPIYWFCQGSSFMGIWSIGHDLGHHAFSEYQWLDDALGFVIHSAF

ctFAD2-5 (1) MGAGGRMNDDATR-------ERDVFKHVPVEKPSFGIADLKKAIPPHCFKRSLTTSFYYRFRDLGLIYAFYYIATKYITHLPQPYS-FVAWPLYWIAQGAICMGLWNIVHDCGHHCFSDYQWLDDTIGFICHSFL

dcACET (1) -----------------------------------------KAIPPHCFEKSLITSFRYLIQDLLMAYALYYVATNYIDQYLPHPLNYLGWAAYIAVQGCVLTGAWVVGHECDHDAFSDYGWINDLVGLIVHSSL

dcDES:OH (1) MGAGGRMS----APSNVKKTETEALRRAPHEKPPFTIGDLKKAIPAHCFEKSLITSFRYLIQDLLMAYALYYVATNYIDQYLPHPINYLGWAAYIAIQGCVLTGAWVVGHECDHDAFSDYGWINDLVGLVVHSSL

fvACET (1) -----------------------------------------KAIPPHCFEKSLITSFRYLIQDLLMAYALYYVATTYIDQYLPHPLNYLGWAAYIAVQGCVLTGAWVVGHECDHDAFSDYGWINDLVGLVVHSSL

hhACET (1) MGAGGRMS----ETPTAKKTAAEALRRAPHEKPPFTIGDLKKAVPAHCFEKSLITSFRYLIQDLLMAYSLYYVATNYID-LLPRPINYLGWAAYIAVQGCVLTGAWVIGHECDHHAFSDYQWVDDLVGLEVHSSL

ctFAD2-8 (1) MGSGVRATE------RSNPESEVVVKRAPSSKPPFTLGDIEKAVSPHWLA-SLVRSSSYLFLDLFLSSLFYYVAAVYIPRLPISLS-YVAWPVYWILQGSVQMGLWVIGHGCGHQAFSDYPWLNDTIGYLLHTGM

ctFAD2-9 (1) MGAGGRSNPR--DLDVKKFDTVEGIKRAPTSKPPFTLGDIKKAIPPHCFKRSLIRSFSYLVYDLTAVSIFYYLATTYIPQLPYPLA-YVAWPVYWFVQGCVFMGLWLIAHECGHHAFSDHVWLEDSIGFVLHSCL

boDES (1) MGGGGRMP---VPTKGKKSKS-DVFQRVPSEKPPFTVGDLKKVIPPHCFQRSVLHSFSYVVYDLVIAALFFYTASRYIHLQPHPLS-YVAWPLYWFCQGSVLTGVWVIAHECGHHAFSDYQWLDDTVGLLLHSAL

ctFAD2-2 (1) MGAGGRMS---NPSEGEKKTELEGIQRVPYQKPPFTVGDVKKAIPPHCFNRSVIRSFSYVVYDLTIASILYYIATTFIPLLPHPLA-YVAWPIYWAVQGCVMTGVWVIAHECGHHAFSDYQWLDDTVGLILHSVL

haDES-2 (1) MGAGGRMS---NPVNGEKKPNPDPLQRVPYQKPPFTVGDVKKAIPPHCFNRSVIRSFSYVVYDLTIASIFYYLANNYIALLPSPLA-YVAWPVYWICQGCVLTGVWVIAHECGHHAFSDYQWLDDTVGLVLHSAL

haDES-3 (1) MGAGGRMS---SPN-GKEKDGPKPLERALHEKPPFTVGDIKKVIPPHCFKRSVIRSFSYVVYDLTIASIFYYLANNYIPLLPNSLA-YVAWPVYWIFQGCVLTGVWVIAHECGHHAFSDYQWLDDTVGLILHSAL

ctFAD2-1 (1) MGGGGCMS----ASETKAEEKKNPLDRVPCAKPPFTISDIKQAIPSHCFNRSLIRSFSYIVYDLAIAFVFYYLATTYIHRLPTPFS-YLAWLAYWIVQGCVLTGAWVVAHECGHHAFSDYQWVDDTVGFIVHSFL

ctFAD2-10 (1) MGGGGCIP----ASKTEAQQT-NHLSRVPYEKPAFTVGDIKRVIPPHCFQRSLIRSFSYVAYDLAIAFLSYHLATTYFHRLSPPLS-YLAWSAYWIVQGCVLTGVWVIAHECGHHAFSDHQWVDDTVGLILHSAL

haDES-1 (1) MGAG---------EYTSVTNENNPLDRVPHAKPPFTIGDLKKAIPPHCFQRSLTRSFSYVLSDLTITAVLYHIATTYFHHLPTPLS-SIAWASYWVVQGCVLTGVWVIAHECGHHAFSDYQWVDDTVGFVLHSSL

ntDES (1) MGAGGNMS----VVTGKTGEKKNPLEKVPTSKPPFTVGDIKKAIPPHCFQRSLVRSFSYVVYDLILVSVFYYIAITYFHLLPSPYC-YLAWPIYWICQGCVCTGIWVIAHECGHHAFSDYQWVDDTVGLILHSAL

oeDES (1) MGAGGRLS----VPATKAEEKKNPLKRVPYLKPPFTVGDIKKTIPPHCFKRSLLRSFSYVVYDLFLVFLFYYIATSYFHLLPSPFS-YLGWSVYWILQGCVCTGVWVIAHECGHHAFSDYQWVDDTVGLILHSTL

siDES (1) MGAGGRMS----DPTTKDEQKKNPLQRVPYAKPPFTLGDIKKAIPPHCFERSVSRSFSYVVYDLVIVFLLYYIATSYFHLLPSPYC-YLAWPIYWAVQGCVCTGIWVIAHECGHHAFSDYQWLDDTVGLILHSAL

ghDES-1 (1) MGAGGRMP--IDGIKEENRGS---VNRVPIEKPPFTLGQIKQAIPPHCFRRSLLRSFSYVVHDLCLASFFYYIATSYFHFLPQPFS-YIAWPVYWVLQGCILTGVWVIAHEWGHHAFRDYQWVDDTVGLILHSAL

ptDES (1) MAANG-----VFASSGKHGGKESRIKRMPHGKPPFTLGKIKKAIPPHCFERSLLRSFSYVVYDLCISFLLCYIAITYIDLLPSPLS-CVAWPMYWILQGSILTGVWVISHECGHHAFSDYRWLDDTVGLILHSAL

rcOH (1) MGGGGRMSTVITSNNSEKKGGSSHLKRAPHTKPPFTLGDLKRAIPPHCFERSFVRSFSYVAYDVCLSFLFYSIATNFFPYISSPLS-YVAWLVYWLFQGCILTGLWVIGHECGHHAFSEYQLADDIVGLIVHSAL

cpDES (1) MGAGGRMS---SPSSIKKSDS-DHVKRVPAAKPPFTLGELKKAIPPHCFHRSVLRSFSYVVYDLTLASIFYYIATTYFQNLPSLLF-YPGWALYWAAQGCVLTGVWVLAHECGHHAFSDYQWLDDTVGLILHSFL

ghDES-4 (1) MGAGGRMS---VPPSQRKQES-GSMKRAPISKPPFTLSEIKKAIPPHCFQRSLIRSFSYLVYDFILVSIFYYVATTYFRNLPQPLS-FVAWPIYWALQGSVLTGVWVIAHECGHHAFSDYQWIDDTVGLILHSSL

ghDES-2 (1) MGAGGRMS---VPTSPKKPEF-NSLKRVPYSKPPFTLSEIKKAIPPHCFQRSVLRSFSYLLYDFILASLFYHVATNYFPNLPQALS-NVAWPLYWAMQGCILTGVWVIAHECGHHAFSDYQWLDDTVGLILHSSL

jcDES (1) MGAGGRMS---VPPSPKKLEA-EVLKRVPYSKPPFTLGQVKKAIPPHCFQRSVLRSFSYVVYDLTLAFIFYYVATNYFHLLPQPLS-YVAWPIYWSLQGCVLTGIWVIAHECGHHAFSDYQWLDDIVGLLLHSCL

luDES (1) MGAGGRMA---VPP-SNKADS-ETFKRSPYSKPPFTLGEIKKAVPPHCFKRSIPRSFSYVAYDLTIAAIFYYIATTYIHLLPNPLS-YVAWPIYWACQGCVLTGVWVLAHECGHHAFSDYQWLDDLVGFVLHSCL

Consensus (1) MGAGGRM LKRVP KPPFTLGDLKKAIPPHCF RSLIRSFSYVVYDLIIAYIFYYLATTYI LLP PLS YLAWPVYW QGCVLTGLWVIGHECGHHAFSDYQWLDDTVGLILHSAL

136 270

atDES (131) LVPYFSWKYSHRRHHSNTGSLERDEVFVPKQKSAIKWYG--KYLNNPLGRIMMLTVQFVLGWPLYLAFNVSGRPYD-GFACHFFPNAPIYNDRERLQIYLSDAGILAVCFGLYRYAAAQGMASMICLYGVPLLIV

lcDES (131) LVPYFSWKYSHRRHHSNTGSLERDEVFVPKQRSAIKWYG--KYLNNPLGRVVMLTIQFVLGWPLYLAFNVSGRPYD-GFASHFFPNAPIYNDRERLQIYISDAGILAVCYGLYRYAAAQGMASMFCLYGVPLLIV

pfOH:DES (132) LVPYFSWKYSHRRHHSNNGSLEKDEVFVPPKKAAVKWYV--KYLNNPLGRILVLTVQFILGWPLYLAFNVSGRPYD-GFASHFFPHAPIFKDRERLQIYISDAGILAVCYGLYRYAASQGLTAMICVYGVPLLIV

plOH (132) LVPYFSWKYSHRRHHANNGSLERDEVFVPPKKAAVKWYV--KYLNNPLGRTVVLIVQFVLGWPLYLAFNVSGRSYD-GFASHFFPHAPIFKDRERLHIYITDAGILAVCYGLYRYAATKGLTAMIYVYGVPLLVV

CoCONJ (126) LTPYFSWKYSHRNHHANTNSLDNDEVYIPKRKSKVKIYS--KLLNNPPGRVFTLVFRLTLGFPLYLLTNISGKKYG-RFANHFDPMSPIFNDRERVQVLLSDFGLLAVFYAIKLLVAAKGAAWVINMYAIPVLGV

haACET (126) YTPYFSWKYSHRNHHANTNSLDNDEVYIPKRKAKVAVYS--KLLNNPPGRVFTLVFRLTLGFPLYLLTNISGKKYG-RFANHFDPLSPIFTERERIQVVISDIGILAVLYATKLLVEAKGAAWVTCMYLIPVLGV

rhACET (94) FTPYFSWKYSHRNHHANTNSLDNDEVYIPKRKAKVAAYS--KLLNXPPGRVFTLVFRLTLGFPLYLLTNISGKKYG-RFANHFDPLSPIFTDRERVQVLLSDLGLLAAFYAIKLLVAAKGFAWVTSMYLIPVMGV

ctFAD2-11 (127) FTPYFAWKYSHRNHHANTNSLDNDEVYIPKRKSKVAIYS--KLLNNPPGRVFTLVFRLTLGFPLYLLTNISGKKYG-RFANHFDPMSPIFTERERIQVLVSDLGILAVLYAIKLAVAAKGAAWVIAMYGIPVVGV

dsACET (95) FTPYFSWKYSHRSHHANTNSLDNDEVYIPKRKSKVRNYS--KILNNPPGRVFTLVFRLTLGFPLYLLTNVSGKKYE-RFANHFDPMSPIFTERERIQVVLSDLGIIAVCYALKVLVAAKGATWVMCMYGVPVIGV

caACET (124) MTPYFSWKYSHRNHHANTNSLDNDEVYIPKSKAKVALYY--KVLNHPPGRLLIMFITFTLGFPLYLFTNISGKKYE-RFANHFDPMSPIFKERERFQVLLSDLGLLAVLYGVKLAVAAKGAAWVTCIYGIPVLGV

cpEPOX (125) LTPYFSWKFSHRNHHSNTSSIDNDEVYIPKSKSKLARIY--KLLNNPPGRLLVLIIMFTLGFPLYLLTNISGKKYD-RFANHFDPMSPIFKERERFQVFLSDLGLLAVFYGIKVAVANKGAAWVACMYGVPVLGV

slEPOX (125) LTPYFSWKYSHRKHHANTNSLENEEVYIPRTQSQLRTYSTYEFLDNTPGRILILVIMLTLGFPLYLLTNVSGKKYD-RFTNHFDPLSPIFTERERIQVALSDLGIVAVFYGLKFLVQTKGFGWVMCMYGVPVIGL

ctFAD2-3 (130) LTPYFGFKYSHRTHHANTNSIEYDEVWIPK-RKSDKLYS--EILNNPLGSFVVFVFKIVLGFPLYFVFNLYGRKYEKGITSHFYPYSPIFNDSERFQIFLTDLGVFGTLYGVYRLALIKGTEWVINFYGMPILFM

ctFAD2-4 (129) LTPYFGFKYSHRTHHANTNSIEYDEVWIPK-RKSDKLYS--EILNNPLGSFVVFVFKIVLGFPLYFVFNLYGRKYEKGITSHFYPYSPIFNDSERFQIFLTDLGVFGTLYGVYRLALIKGTEWVINFYGMPILFM

ctFAD2-7 (125) LTPYFSFKYSHRSHHAHTNSIEYDEVYIPK-RKADTFYS--EFLNNGPGNVFTLLLRTTLGLPLYLIFNTYGRDYN-GFANHFLPQSGIFNDSERAQVVLSDVGIFAVLYALYRLVLIQGLKSTIFLQGIPLFVM

ctFAD2-6 (127) LTPYFSFKYSHRSHHAHTNSMEYDEVWIPK-RKADTMYS--EVLNNPLGNLFMTVVRLLFSFPMYFTFNIHGRPYN-GFVSHFYPQSPMFNDSERKLVWLSDAGMVAAFYGLYKIAQSTSATWLFCIYGAPLLVM

ctFAD2-5 (128) LTPYFSFKYSHRTHHANTSSLERDEVWVPK-RKHDTWFY--EVLSNPVGSFIMLVFRLFFGFPLYFMFNLHGRIYK-GFPSHFNPLGPIFNDRERANIWLSDAGVLTVVYALYRIGAKEGLQWVLFVYIYPLMAM

dcACET (95) LVPYFSWKISHRRHHANTQSLENDEVYVPRFKSNIRNYY--KIFNNPPGRVLVWVTTLLIGFPLYLMFNVSGHKYE-RWTSHCDPHSPLYTERERKQIIVSDVAILAVIYGLYNLVLAKGFVWVFCVYGGPLLVV

dcDES:OH (132) MVPYFSWKISHRRHHANTQSLENDEVYVPRFKSNIRNYY--KILNNPPGRVIVWLITLLIGFPIYLMFNVSGHKYE-RWTSHYDPHSPLYTERERKQIIVSDFAILAVLYGLYNLVLAKGFAWVFCVYGGPLLVV

FvACET (95) MVPYFSWKISHRRHHANTQSLENDEVYVPRFRYNIRNYY--KVLNNPLGRVLVWVTTLLIGFPLYLMFNVSGHKYE-RWTSHYDPHSPLYTERERKQIIVSDIAILAVIYGLYRLVLIKGFAWVFCVYGGPLLVV

HhACET (131) LVPYFSWKISHRRHHANTQSLENDEVYVPRFKSNIRNYY--KILNNPPGRVFVWLSTLLIGFPLYLMFNVSGHKYE-RWTSHYDPHSPLYSDRERKEIIISDVAILTVIYGLYRLVLAKGFEWVFLVYGGPLLVV

ctFAD2-8 (128) PAPYFSWKYSHRRHHSNTGSLEHDESFVPKKKSSLNSIA--RMLNNPPGRLLRLLTLCTIGWLLYICFNVSGRKYE-KFANHFDPKSPIYNDRERFQILLTDIGLLVTSYGLYKLALAQGFAWLITIYFAPLVIV

ctFAD2-9 (133) LTPYFSWKISHRRHHANTGSLEHDEVYVPKTKAKLGASA--FYLDNPIGRTLTLMVKLTLGWYIYLAINAAGRPYE-KFASHYDPRSEMFSDNERVLILMSDIGLLSFSFLLYKVAMVQGFAWVFCVYGGALMVM

boDES (131) LVPYFSWKYSHRRHHSNTGSLERDEVFVPKKRSGISWSS--EYLNNPPGRVLVLLVQLTLGWPLYLMFNVSGRPYD-RFACHFDPKSPIYNDRERLQIYISDAGIVAVMYGLYRLVAAKGVAWVVCYYGVPLLVV

ctFAD2-2 (132) LVPYFSWKYSHRRHHSNTGSIEHDEVFVPKLKSGVRSTA--KYLNNPPGRILTLLVTLTLGWPLYLMFNVSGRYYD-RFACHFDPNSPIYSNRERAQIFISDAGIFAVLYGLYRLAAVKGLVWVLTVYAGPLLVV

haDES-2 (132) LVPYFSWKYSHRRHHSNTGSIEHDEVFVPKLKSGVRSTA--RLLNNPPGRILTLLVTLTMGWPLYLMFNVSGRYYD-RFACHFDPNSPIYSNRERAQIFISDAGILTVLFVLFRVAMTKGLTWVLTMYAGPLLVV

haDES-3 (131) LVPYFSWKYSHRRHHSNTGSIEHDEVFVPKLKSSVRSTA--KYLNNPPGRILTLLVTLTMGWPLYLMFNVSGRYYD-RFACHFDPNSPIYSNRERAQIFISDAGILTVFYILFRLASTKGLVWVLTMYGGPLLVV

ctFAD2-1 (131) LVPYFSWKYSHRRHHSNTASLERDEVFVPKPRSKLPWYS--KYLNNPPGRIISLFATLTLGWPLYLSFNVSGRPYD-RFACHYAPNSPIYNHRERLQIWLSDVGIVAMAFVLYRVALVKGVSWVVCVYGIPLLIV

ctFAD2-10 (130) LVPYFSWKCSHRRHHLNTASLERDEVFVPKPKSKIPWYS--KYLNNPPGRLIIVFTTLVLGWPLYLAFNVSGRPYD-RFACHYAPNSPIFTNRERLQIWISDAGIIAVSYLLYRMALAKSVAWVVCIYGVPLLIV

haDES-1 (126) LVPYFSWKYSHHRHHSNTGSLERDEVFVPKSRSKVPWYS--KYFNNTVGRIVSMFVTLTLGWPLYLAFNVSGRPYD-RFACHYVPTSPMYNERKRYQIVMSDIGIVITSFILYRVAMAKGLVWVICVYGVPLMVV

ntDES (131) MVPYFSWKYSHRRHHSNTGSLERDEVFVPKPKSQLGWYS--KYLNNPPGRVMSLTVTLTLGWPLYLAFNVSGRHYD-RFACHYDPYGPIYNDRERLQIFLSDAGVLGAGYLLYRIALVKGLAWLVCMYGVPLLIV

oeDES (131) LVPYFSWKYSHRRHHSNTGSLERDEVFVPKPKSKLSWFT--KYLNNPPGRVMTLVITLTLGWPLYLALNVSGRPYD-RFACHYDPHGPIYNDRERLQIYISDVCVIATSYILYRVALAQGLVWLTCVYGVPLLIV

siDES (131) LVPYFSWKYSHRRHHSNTGSLERDEVFVPKPKSRVSWYS--KYLNNPLGRVITLVVTLTLGWPLYLLFNVSGRPYN-RFACHFDPYGPIYNDRERLQIFISDAGIIAAVCVLYRVALVKGLAWLVCVYGVPLLIV

ghDES-1 (130) LVPYFSWKISHRRHHSNTGSMERDEVFVPKPKSKLSCFA--KYLNNPPGRVLSLVVTLTLGWPMYLAFNVSGRYYD-RLASHYNPYGPIYSDRERLQVYISDTGIFAVIYVLYKIAATKGLAWLLCTYGVPLLIV

ptDES (130) LVPYFSWKYSHRRHHSNTGSLERDEVFVPKPKSRIAWYS--KYLNNPPGRALSLVVTLLLGWPLYLAFNVSGRPYD-RFACHYDPYGPIYSDRERLQIYISDLGIFAATFVLYSIAVSRGLAFLICIYGVPLLIA

rcOH (135) LVPYFSWKYSHRRHHSNIGSLERDEVFVPKSKSKISWYS--KYSNNPPGRVLTLAATLLLGWPLYLAFNVSGRPYD-RFACHYDPYGPIFSERERLQIYIADLGIFATTFVLYQATMAKGLAWVMRIYGVPLLIV

cpDES (131) MVPYFSWKYSHRRHHSNTGSLERDEVFVPKKKSALKWYS--PYLNNPLGRVLTLTITLVLGWPLYLAFNVSGRPYD-RFACHYDPYGPIYTDRERLQIFISDAGLLAVSYGLYRLVLAKGLAWVVCVYGVPLLIV

ghDES-4 (131) LVPYFSWKYSHRRHHSNTGSLERDEVFVPKKRSSIRWWA--KYLNNPPGRFVTITIQLTLGWPLYLAFNVAGRPYE-GFACHYNPYGPIYNDRERLQIYISDVGVLAVTYGLYRLVLAKGLAWVICVYGVPLLIV

ghDES-2 (131) LVPYFSWKYSHRRHHSNTGSLERDEVFVPKKKSGLRWWA--KHFNNPPGRFLSITIQLTLGWPLYLAFNVAGRPYD-RFACHYDPYGPIFSDRERLQIYISDAGVLAVAYALYRLVLAKGVGWVISVYGVPLLVV

jcDES (131) LVPYFSWKHSHRRHHSNTGSLERDEVFVPKKKSNIRWFS--KYLNNLPGRLFTLTITLALGWPLYLAFNVSGRHYD-RFACHFDPYGPIYNDRERTEIFISDAGVLAVTYGLYRLALAKGFAWVICVYGVPLLVV

luDES (130) MVPYFSWKHSHRRHHSNTGSLERDEVFVPKQKSAIGWHS--KYLNNPPGRVLTLAVTLTLGWPLYLAFNVSGRPYD-RFACHYDPKSPIYNDRERTEIFFSDAGILAVSFALYKLAVAKGLAWVVCVYGVPLLVV

Consensus (136) LVPYFSWKYSHRRHHSNTGSLE DEVFVPK KS I YS KYLNNPPGRVLTLVV LTLGWPLYL FNVSGR YD RFA HFDP SPIY DRERLQIFISDLGILAV YGLYRLALAKGLAWVICVYGVPLLVV

271 396

atDES (263) NAFLVLITYLQHTHPSLPHYDSSEWDWLRGALATVDRDYG-ILNKVFHNITDTHVAHHLFSTMPHYNAMEATKAIKPILGDYYQFDGTPWYVAMYREAKECIYVEPDRE-GD--KKGVYWYNNKL-

lcDES (263) NFFLVLITYLQHTHPSLPHYDSSEWDWLRGALATVDRDYG-ILNKVFHNITDTHVAHHLFSTMPHYNAMEATKAIKPILGEYYQFDGTPWYKAMYREAKECIYVEPDRE-DE--KKGVYWYNNKL-

pfOH:DES (264) NFFLVLVTFLQHTHPSLPHYDSTEWEWIRGALVTVDRDYG-ILNKVFHNITDTHVAHHLFATIPHYNAMEATEAIKPILGDYYHFDGTPWYVAMYREAKECLYVEPDTE-RG--KKGVYYYNNKL-

plOH (264) NFFLVLVTFLQHTHPSLPHYDSTEWDWIRGAMVTVDRDYG-ILNKVFHNITDTHVAHHLFATIPHYNAMEATEAIKPILGDYYHFDGTPWYVAMYREAKQCLYVEQDTE-K---KKGVYYYNNKL-

coCONJ (258) SVFFVLITYLHHTHLSLPHYDSTEWNWIKGALSTIDRDFG-FLNRVFHDVTHTHVLHHLISYIPHYHAKEARDAIKPVLGEYYKIDRTPIFKAMYREAKECIYIEPDED---SEHKGVFWYHKM--

haACET (258) HMFFVLITYLHHTHLSLPHYDSSEWNWIRGALSTIDRDFG-FLNRVFHDVTHTHVLHHLISYIPHYHAKEARDAIKPVLGEFYKIDRTPIFKAMWREAKECIYIEPDED---SEHKGTYWYHKM--

rhACET (226) HMFFVLITYLHHTHLSLPHYDSTEWNWIKGALSTIDRDFG-FLNRVFHDVTHTHVLHHLISYIPHYHAKEARDAIKPVLGEFYKIDRTPIFKAMWRXAKEXV------------------------

ctFAD2-11 (259) HVFFVLITYLHHTHLSLPHYDSTEWDWIRGALSTIDRDFG-FLNRVFHDVTHTHVLHHLISYIPHYHAKEARDAIKPILGEYYKIDRTPIFKAMWREAKESIYIEPDEN---SEHKGTYWYHKEL-

dsACET (227) HAFFVLITYLHHTHLSLPHYDSSEWNWIKGALSTIDRDFG-FLNRVFHDVTHTHVLHHLISYIPHYHAKEARDAIIPVLGEFYKIDRTPIFKAMWREAKEC-------------------------

caACET (256) FIFFDIITYLHHTHLSLPHYDSSEWNWLRGALSTIDRDFG-FLNSVLHDVTHTHVMHHLFSYIPHYHAKEARDAINTVLGDFYKIDRTPILKAMWREAKECIFIEPEKG---RESKGVYWYNKF--

cpEPOX (257) FTFFDVITFLHHTHQSSPHYDSTEWNWIRGALSAIDRDFG-FLNSVFHDVTHTHVMHHLFSYIPHYHAKEARDAIKPILGDFYMIDRTPILKAMWREGRECMYIEPDS-----KLKGVYWYHKL--

slEPOX (259) NSFIIVITYLHHTHLSSPHYDSTEWNWIKGALTTIDRDFG-LLNRVFHDVTHTHVLHHLFPYIPHYHAKEASEAIKPILGDYRMIDRTPFFKAMWREAKECIYIEQDAD---SKHKGTYWYHKM--

ctFAD2-3 (262) SGFFILLTYLHHTHPSIPHYDSTEWDWLRGALATVDRNFG-FLNHAFHDVTRTHAVHHLFPTIPHYHTFEARQAVMPILGDYYKYDDTPILEAVWRETKDCIFIEPEEVNGE--KKGIYWFYK---

ctFAD2-4 (261) SGFFILLTYLHHTHPSIPHYDSTEWDWLRGALATVDRNFG-FLNHAFHDVTRTHAVHHLFPTIPHYHTFEARQAVMPILGDYYKYDDTPILEAVWRETKDCIFIEPEEVNGE--KKGIYWFYK---

ctFAD2-7 (256) SGFFIFLTYLNHTHPAIAHYDSTEWDWLRGALSTIDRDFG-ILNRVFHNANHTHGIHHLFPTIPHYHAIEAREAVKPILGDYYMYDDTPILKAMWRDTKECIYVEPDDE-----KKGVYWYFK---

ctFAD2-6 (258) NAHFIFFTFLHHSHVSLAHYDSREWDWIRGALSTVDRNYG-ILNTVFHDVTCAHVVHHLISTIPHYHTVEATNAVKPILGDYYKYDDTPILKAFWRETKNCIYVEPDEG-AE--DSGVYWFRR---

ctFAD2-5 (259) SAFFIMFTYLHHTHPSIAHYDSSEWDWLRGALSTVDRDYG-ILNNIFHDVTSAHVVHHLFSSIPHYNTVEATQYIKPILGEYYNYDYTPILKAIWRDTKECLFIEEDPE-----KKGVYWFHK---

dcACET (227) NGWFTLITILNHTHPSLPYYDSTEWDWLRGALCTVDRDYG-ILNKVFHNVCNAHVCHHIFSMIPHYHGLEATEAMKPLLGDYYQYDGTPILKAMYREMK---------------------------

ccDES:OH (264) NGWFTLITILNHTHPSLPYYDSSEWDWLRGALCTVDRDYG-ILNKVFHNVCNAHVCHHIFSMIPHYHGLEATEAMKPLLGDYYQYDGTPILKAMYREMKECIYVEKDEG----ETKGVYWSRKDI-

fvACET (227) NMWFTLITILNHTHPSVPYYDSTEWDWLRGALCTVDRDYG-ILNKVFHNVCNAHVCHHIFSMIPHYHGLEATEAMKPVLGDYYQYDGTPILKAMYREMK---------------------------

hhACET (263) NGWFVLITILNHTHPSLPYYDSTEWDWLRGALCTVDRDYG-ILNKVFHNVCNAHVCHHIFSMIPHYHGLEATQAMKPILGEYYQYDGTPILKAMYREMKECIYVEKDEG----ETKGVYWYRSEF-

ctFAD2-8 (260) YGFLVVITWLHHTHRSLPHYDSTEWSWLRGALSTMDRDYG-VFNTVLHHVTDTHVVHHFFFTIPHYHAMEATKSIKPFLGEYYQFDDTPIIKAMWREATECFFVEADEGEGK--SKGVYWFNNKM-

ctFAD2-9 (265) NAFLVTITYLHHTHPSLPHYDDSEWNWMKGAFATVDRDYGVVLNKVFHNITDTHVLHHLFSYIPHYHAMEATKAIRPVLGEFYQIDRTPFFVALWRESKSCLFIEPDESDEK--NKGIYWYRSKY-

boDES (263) NGFLVLITYLQHTQPSLPHYDSSEWDWLKGALATVDRDYG-FLNKVLHNITDTHVAHHLFSTMPHYHAMEATKAIKPILGDYYQCDRTPVFKAMYREVKECIYVEADEGDN---KKGVFWYKNKL-

ctFAD2-2 (264) NGFLVLITFLQHTHPSLPHYDSTEWDWLRGALATIDRDYG-ILNKVFHNITDTHVTHHLFSTMPHYHAMEATKAIIPILGDYYQFDGTSVFKAMYRETKECIYVDKDEEV----KDGVYWYRNKI-

haDES-2 (264) NGFLVLITFLQHTHPSLPHYDSTEWDWLRGALATIDRDYG-VLNKVFHNITDTHVAHHLFSTMPHYHAMEATKVIKPILGEYYQFDGTSIFKAMYRETKECIYVDKDEEV----KDGVYWYRNKI-

haDES-3 (263) NGFLVLITFLQHTHPSLPHYDSTEWDWLRGALATVDRDYG-ILNKVFHNITDTHVTHHLFSTMPHYHAMEATKAIKPILGDYYQFDGTSIFKAMYRETKECIYVDKDEDV----KDGVYWYRNKI-

ctFAD2-1 (263) NGFLVLITFLQHTHPSLPHYDGSEWDWLRGALATVDRDYG-VLNKVFHNITDTHVVHHLFSTMPHYHAMEATRAVKGLLGEYYQFDETPFYAAMWREAKECLFVEADE--G---KGGVFWYKNK--

ctFAD2-10 (262) NGFLVMITYLQHTHPSLPHYDDSEWDWLRGALATVDRDYG-LLNKVFHNITDTHVVHHLFSTMPHYHAMEATKAVEPVLGGYYRFDDTPFYVAMWREAKECLYVETDE--K---KGGVFWYKNKY-

gaDES-1 (258) NAFLVLITYLQHTHPGLPHYDSSEWEWLKGALATVDRDYG-VLNKVFHHITDTHVVHHLFSTMPHYNAMEAQKALRPVLGEYYRFDKTPFYVAMWREMKECLFVEQDDE-G---KGGVFWYKNKMN

ntDES (263) NGFLVLITYLQHTHPSLPHYDSSEWDWLRGALATVDRDYG-ILNKVFHNITDTHVVHHLFSTMPHYNAMEATKAVKPLLGDYYQFDGTPVFKAMWREAKECIYVEKDEASQ---GKGVFWYKNKL-

oeDES (263) NGFLVLITYLQHTHPPLPHYDSSEWDWLRGALATVDRDYG-VLNNVFHNITDTHVAHHLFSTMPHYHAMEATKAIKPLLGEYYQSDGTPFYKAMWREAKECLYVEPDE--P---NKGVFWYKNKF-

siDES (263) NGFLVLITFLQHTHPSLPHYDSSEWDWLRGALATVDRDYG-VLNKVFHNITDTHVTHHLFSTMPHYHAMEATKAIKPILGQYYQFDGTPFYKAMWREAKECLYVEPDESTP---DKGVFWYKNKF-

ghDES-1 (262) NAFLVLITYLQHTHSALPHYDSSEWDWLRGALSTMDRDFG-VLNKVFHNITDTHVAHHLFSTMPHYHAMEATKAIKPILGKYYPFDGTPIYKAMWREAKECLYVEPDVGGGGGGSKGVFWYRNKF-

ptDES (262) NGFLVTITYLQHTHPALPHYDSSEWEWLRGALATMDRDYG-ILNKVFHNITDTHVAHHLFSNIPHYHAMEATKAIKPILGEFYQFDDTPIYKALWREAKECLYVEPDDGAP---EKGVFWYRNEF-

rcOH (267) NCFLVMITYLQHTHPAIPRYGSSEWDWLRGAMVTVDRDYG-VLNKVFHNIADTHVAHHLFATVPHYHAMEATKAIKPIMGEYYRYDGTPFYKALWREAKECLFVEPDEGAP---TQGVFWYRNKY-

cpDES (263) NGFLVLITYLQHTHPALPHYDSSEWDWLRGALATVDRDYG-ILNKVFHNITDTHVAHHLVSTMPHYHAMEATKAIKPILGEYYHFDGTPFVKAMWREAKECIYVEPDEG-EK--TKGVFWYNNKF-

ghDES-4 (263) NAFLVMITYLQHTHPALPHYDSSEWDWLRGALATVDRDYG-ILNKVFHNITDTHIAHHLFSTMPHYHAMEATKAIKPILGEYYSFDGTPVYKAIFREAKECIYVEPDEGEQS--SKGVFWFRNKI-

ghDES-2 (263) NAFLVMITYLQHTHPSLPHYDSSEWDWMRGALSTVDRDYG-ILNKVFHNITDTHVAHHLFSTMPHYHAMVATKAIKPILGEYYQFDGMPVYKAIWREAKECLYVEPDEG-DK--DKGVFWFRNKL-

jcDES (263) NAFLVMITYLQHTHPSLPHYDSSEWDWLRGALATVDRDYG-ILNKVFHNITDTHVAHHLFSTMPHYHAMEATNAIKPILGEYYQFDRTPFFKAMWREAKECIYVEPDDA-DQ--SRGVFWYKNKF-

luDES (262) NGFLVLITFLQHTHPSLPHYKSSEWDWLRGALATMDRDYG-FLNTVFHNITDTHVAHHLFSTMPHYHAMEATKAIKPVLGEYYQFDGTPFIKAMWREAKECVYVEPDEG-DQ--NKGVFWYNNKL-

Consensus (271) NGFLVLITYLQHTHPSLPHYDSSEWDWLRGALATVDRDYG ILNKVFHNITDTHV HHLFSTIPHYHAMEATKAIKPILGEYYQFD TPIYKAMWREAKECIYVEPDE KGVYWY NK

Additional file 2_Figure S2. Mass spectral identification of DMOX derivatives of C18:2^Δ9(Z),12(E)^ and C18:2^Δ9(Z),12(Z)^ from *S. cerevisae* expressing safflower CtFAD2-11.

Additional files 3_Figure S3. Mass spectral identification of DMOX derivatives of crepenynic acid (9-octadecen-12-ynoic acid) from *N. benthamiana* leaves transiently expressing safflower CtFAD2-11 (A). methyl crepenynate standard.

Additional file 4_Table S1. Oligonucleotide primers used in the 3’RACE of multiple *CtFAD2* genes in safflower

| Primer gene | Sense sequence | Antisense sequence |
| --- | --- | --- |
| *CtFAD2-3* | 5'- CTTCAGCGAGTACCAATGGCTCGAC-3' | 5’- GGTTTCATCGTCCACTCCTTGA -3' |
| *CtFAD2-4* | 5’- CTTCAGCGAGTACCAATGGCTCGAC-3’ | 5’- GGTTTCATCGTCCACTCCTTGA -3' |
| *CtFAD2-5* | 5’- ATGACACCATTGGCTTCATCTGCCA -3' | 5’- CTTTCTGCTCACTCCATACTTC -3' |
| *CtFAD2-6* | 5’- AGCGAATATCAGTGGCTTGACGATG -3' | 5’- ACTCCGCTTTCCTCACTCCGTAC -3' |
| *CtFAD2-7* | 5’- CATGAATGTGGTCATCATGCCTTTAG -3' | 5’- CTTCTTCATCCATTCGGTTTGC -3' |
| *CtFAD2-8* | 5’- CGTGGTTGAATGACACCATTGGTTAC -3' | 5’- ACCTTCTACACACCGGTATGCCT -3' |
| *CtFAD2-9* | 5’- CATGGAAGATAAGCCACCGTCGACATC -3’ | 5’- AACACGGGTTCGCTTGAGCACGA -3' |
| *CtFAD2-10* | 5’- TGCATACCCGCAAGCAAAACCG -3’ | 5’- CCATCTCTCGAGAGTTCCTTAC -3' |
| *CtFAD2-11* | 5’- ATGTGGTCACCATGCCTTTAGTGAG -3’ | 5’- TGGAATGGTCCTCCATTCCGCTC -3' |

Additional file 5_Table S2. Oligonucleotide primers used for amplification of the entire coding region of *CtFAD2* genes in safflower

| Primer gene | Sense sequence | Antisense sequence |
| --- | --- | --- |
| *CtFAD2-1* | 5’- TGAAAGCAAGATGGGAGGAGG -3' | 5’- TCACAACTTTACTTATTCTTGT -3' |
| *CtFAD2-2* | 5’- ATTGAACAATGGGTGCAGGC -3' | 5'CATCATCTTCAAATCTTATTC -3' |
| *CtFAD2-3* | 5’- AATCAGCAGCAGCACAAGC -3' | 5’- CAAACATACCACCAAATGCTACT -3' |
| *CtFAD2-4* | 5’- CTCAGTAACCAGCCTCAAAACTTG -3' | 5’- GCGGATTGATCAAATACTTGTG -3' |
| *CtFAD2-5* | 5’- ATCACAGGAAGCTCAAAGCATCT -3' | 5’- GTAGGTTATGTAACAATCGTG -3' |
| *CtFAD2-6* | 5’- TGAAGACGTTAAGATGGGAGCTG -3’ | 5’- GTAGGTTATGTAACAATCGTG -3' |
| *CtFAD2-7* | 5’- CAGATCCAACACTTCACCACCAG -3’ | 5’- AGATCTAAAGAATTTCCATGGTG -3' |
| *CtFAD2-8* | 5’- CTGCTCTCTACGACACTAAATTCAC -3’ | 5’- TCTATCTAATGAGTATCAAGGAAC -3' |
| *CtFAD2-9* | 5’- CTGAATTCACACCCACAGATAGCTAG -3’ | 5’- ACATCCCTTCTTAGCTTTAACTA-3' |
| *CtFAD2-10* | 5’- ACTTCGCCCTCTGTTATCTGG -3’ | 5’- CCATACACATACATCCTACACGAT -3' |
| *CtFAD2-11* | 5’- ACTCACAATAACTTCATCTCTCTC -3’ | 5’- CTACTAGCCATACAATGTCTTCG -3' |

Additional file 6_Table S3. Oligonucleotide primers used for the amplification of 5’UTR intron of *CtFAD2* genes in safflower.

| Prmier gene | Sense sequence | Antisense sequence |
| --- | --- | --- |
| *CtFAD2-1* | 5’- GAGATTTTCAGAGAGCAAGCGCTT -3' | 5’- CTTTGGTCTCGGAGGCAGACATA -3' |
| *CtFAD2-2* | 5’- CAAAAGGAGTTTCAGAAAGCCTCC -3' | 5’- ACTCGTTGGATGCCTTCGAGTTC- 3' |
| *CtFAD2-3* | 5’- AATCAGCAGCAGCACAAGC -3’ | 5’- AAGGCGGTGACAATTATGATATC -3' |
| *CtFAD2-4* | 5’- CTCAGTAACCAGCCTCAAAACTTG -3’ | 5’- AAGGCGGAGACGATTATGATATC -3' |
| *CtFAD2-5* | 5’- ATCACAGGAAGCTCAAAGCATCT -3’ | 5’- ATCATCTCTTCGGTAGGTTATG -3' |
| *CtFAD2-7* | 5’- CAGATCCAACACTTCACCACCAG -3’ | 5’- CTAAAGAATTTCCATGGTGTTAC -3' |
| *CtFAD2-10* | 5’- ACTTCGCCCTCTGTTATCTGG -3’ | 5’- GAGAGACGGTGGAAGTAGGTG -3' |
| *CtFAD2-11* | 5’- CTCACAATAACTTCATCTCTCTC -3’ | 5’- AAAGACATAGGCAACAACGAGATC -3' |

Additional file 7_Table S4. Oligonucleotide primers used for RT-qPCR in the expression profile study of safflower *CtFAD2* genes

| Primer gene | Sense sequence | Antisense sequence |
| --- | --- | --- |
| *CtFAD2-1* | 5’- GTGTATGTCTGCCTCCGAGA -3’ | 5’- GCAAGGTAGTAGAGGACGAAG -3' |
| *CtFAD2-2* | 5’- GCCTCCAAAGATTCATTCAGGTC - 3' | 5’- CAAGATGGATGCGATGGTAAGG -3' |
| *CtFAD2-3* | 5’- ACGTGGCGGTCTCAGGTT -3' | 5’- AGGCGGTGACAATTATGATATC -3' |
| *CtFAD2-4* | 5’- AAGGCAGGCCGTGATGCCGAT -3’ | 5’- AGTATTTGATCAATCCGCTGG -3' |
| *CtFAD2-5* | 5’- CAATACGGTAGAGGCCACACAG -3' | 5’- ATCATCTCTTCGGTAGGTTATG -3' |
| *CtFAD2-6* | 5’- GACATGTGCTCACGTGGTGCAT -3' | 5’- GTTGCTAATATCCACACCCTA -3' |
| *CtFAD2-7* | 5’- CGAATCACACCCACGGGATC -3’ | 5’- CTAAAGAATTTCCATGGTGTTAC -3' |
| *CtFAD2-8* | 5’- GAGCAACGGAGAGAAGTAACC -3’ | 5’- GAGGGATGATAGAAAGAGGTCC -3' |
| *CtFAD2-9* | 5’- CATGTGTGGCTGGAGGATTCGA -3’ | 5’- GCACCGAGTTTAGCCTTTGTCT -3' |
| *CtFAD2-10* | 5’- CCAACAAACAAACCATCTCTCG -3’ | 5’- GAGAGACGGTGGAAGTAGGTG -3' |
| *CtFAD2-11* | 5’- CCATTGATCCACCCTTCACCTTA -3' | 5’- AAAGACATAGGCAACAACGAGATC -3' |
| *KASII* | 5’- CTGAACTGCAATTATCTAGG -3’ | 5’- GGTATTGGTATTGGATGGGCG -3' |
